# Analysis of the splicing landscape of the frontal cortex in FTLD-TDP reveals subtype specific patterns and cryptic splicing

**DOI:** 10.1007/s00401-025-02901-7

**Published:** 2025-06-06

**Authors:** Júlia Faura, Bavo Heeman, Cyril Pottier, Matthew C. Baker, Mariely DeJesus-Hernandez, Fahri Küçükali, Laura Heiß, Sarah Wynants, Marleen Van den Broeck, Peter De Rijk, Tim De Pooter, Geert Joris, NiCole A. Finch, Yan Asmann, Mojca Strazisar, Melissa E. Murray, Leonard Petrucelli, Björn Oskarsson, Kristel Sleegers, Keith A. Josephs, Aivi T. Nguyen, R. Ross Reichard, Ronald C. Petersen, Bradley F. Boeve, Neill R. Graff-Radford, Dennis W. Dickson, Marka van Blitterswijk, Rosa Rademakers

**Affiliations:** 1 Applied and Translational Neurogenomics, VIB Center for Molecular Neurology, VIB, Antwerp, Belgium; 2 Department of Biomedical Sciences, University of Antwerp, Antwerp, Belgium; 3 Department of Neuroscience, Mayo Clinic, Jacksonville, FL USA; 4 Department of Neurology, Washington University School of Medicine, St Louis, MO USA; 5 NeuroGenomics and Informatics Center, Washington University School of Medicine, St Louis, MO USA; 6 Complex Genetics of Alzheimer’s Disease Group, VIB Center for Molecular Neurology, VIB, Antwerp, Belgium; 7 Neuromics Support Facility, VIB Center for Molecular Neurology, VIB, Antwerp, Belgium; 8 Department of Quantitative Health Sciences, Mayo Clinic, Jacksonville, FL USA; 9 Department of Neurology, Mayo Clinic, Jacksonville, FL USA; 10 Department of Neurology, Mayo Clinic, Rochester, MN USA

**Keywords:** Frontotemporal dementia, TDP-43, Splicing, Transcriptomics

## Abstract

Dysregulation of TDP-43 as seen in TDP-43 proteinopathies leads to specific RNA splicing dysfunction. While discovery studies have explored novel TDP-43-driven splicing events in induced pluripotent stem cell (iPSC)-derived neurons and TDP-43 negative neuronal nuclei, transcriptome-wide investigations in frontotemporal lobar degeneration with TDP-43 aggregates (FTLD-TDP) brains remain unexplored. Such studies hold promise for identifying widespread novel and relevant splicing alterations in FTLD-TDP patient brains. We conducted the largest differential splicing analysis (DSA) using bulk short-read RNAseq data from frontal cortex (FCX) tissue of 127 FTLD-TDP (A, B, C, *GRN* and *C9orf72* carriers) and 22 control subjects (Mayo Clinic Brain Bank), using Leafcutter. In addition, long-read bulk cDNA sequencing data were generated from FCX of 9 FTLD-TDP and 7 controls and human *TARDBP* wildtype and knock-down iPSC-derived neurons. Publicly available RNAseq data (MayoRNAseq, MSBB and ROSMAP studies) from Alzheimer’s disease patients (AD) was also analyzed. Our DSA revealed extensive splicing alterations in FTLD-TDP patients with 1881 differentially spliced events, in 892 unique genes. When evaluating differences between FTLD-TDP subtypes, we found that *C9orf72* repeat expansion carriers carried the most splicing alterations after accounting for differences in cell-type proportions. Focusing on cryptic splicing events, we identified *STMN2* and *ARHGAP32* as genes with the most abundant and differentially expressed cryptic exons between FTLD-TDP patients and controls in the brain, and we uncovered a set of 17 cryptic events consistently observed across studies, highlighting their potential relevance as biomarkers for TDP-43 proteinopathies. We also identified 16 cryptic events shared between FTLD-TDP and AD brains, suggesting potential common splicing dysregulation pathways in neurodegenerative diseases. Overall, this study provides a comprehensive map of splicing alterations in FTLD-TDP brains, revealing subtype-specific differences and identifying promising candidates for biomarker development and potential common pathogenic mechanisms between FTLD-TDP and AD.

**Supplementary Information:**

The online version contains supplementary material available at 10.1007/s00401-025-02901-7.

## Introduction

TAR DNA-binding protein 43 (TDP-43) is a DNA- and RNA-binding protein involved in fundamental RNA processing activities including RNA transcription, splicing, and transport. It binds to thousands of pre-mRNA/mRNA targets, with a high affinity for GU-rich sequences and long intron-containing transcripts [37]. Under normal physiological conditions, TDP-43 is present in the nucleus, but under pathological conditions, nuclear and cytoplasmic aggregates of aberrantly phosphorylated, ubiquitinated, and cleaved fragments of TDP-43 are observed in the central nervous system and the muscle fibers [11]. This accumulation is a common pathological feature of diverse neurodegenerative diseases such as amyotrophic lateral sclerosis (ALS), frontotemporal lobar degeneration (FTLD), Alzheimer’s disease (AD), and limbic predominant age-related TDP-43 encephalopathy (LATE) [29].

FTLD is a clinical, pathological, and genetically heterogeneous disease that represents 10–20% of all dementias. It is clinically relevant because of its early age at onset, especially compared to AD, and its dramatic impact on core human qualities, including personality, insight, and verbal communication [55]. FTLD with TDP-43 aggregation (FTLD-TDP) is the most common FTLD subtype, accounting for ~ 50% of the cases. TDP-43 accumulates in the form of insoluble and cytoplasmic or neuritic aggregates in neurons and glia of the frontotemporal region of FTLD patients’ brains [23]. These aggregates show heterogeneity in both distribution and morphology, leading to an established classification system defining distinct pathological FTLD-TDP subtypes, with the most common ones being FTLD-TDP type A, B and C [44]. While some clinical phenotypes are more likely to occur in a specific FTLD-TDP subtype, a strong clinicopathological correlation is lacking. On the other hand, some causal genes were found to consistently lead to specific FTLD-TDP subtypes. Loss-of-function mutations in the progranulin gene (*GRN*) always lead to FTLD-TDP type A and repeat expansions in *C9orf72* lead to type A and type B, but the etiology of a significant proportion of FTLD-TDP cases remains unexplained.

In vitro and in vivo studies have revealed that loss of the normal TDP-43 function leads to an increase in the inclusion of cryptic exons (CE), which are normally repressed but under these conditions are aberrantly included in the mRNAs, and to the skipping of constitutive exons [24, 39]. In recent years, two CE inclusions in *STMN2* and *UNC13A* have been well-characterized in TDP-43 proteinopathies [13, 42, 61], and since these discoveries, efforts have focused on identifying additional TDP-43-driven splicing events due to their potential as biomarkers and therapeutic targets [46]. Most notably, Seddighi et al. demonstrated the translation of peptide products from 13 novel cryptic events [63]. One of these cryptic peptides, HDGFL2, showed potential as a diagnostic marker for TDP-43 pathology, since its cryptic neoepitope has been detected in blood and CSF [27].

For the discovery of TDP-43-driven cryptic events, most studies have used in vitro models, such as TDP-43 knock-down induced pluripotent stem cells (iPSC)-derived neurons [13, 42, 47, 63]. However, these models do not fully reflect the actual processes occurring in human tissue. In vivo, certain splicing events may be more common than others, which in vitro models might fail to model. In addition, neurons are not the only brain cell type affected by TDP-43 pathology, oligodendrocytes and astrocytes also show TDP-43 accumulation in their cytoplasm [38, 51].

Here, we took advantage of the human disease model to identify those alternatively spliced events most abundant in diseased brains. In doing so, we further aimed to reveal the full brain splicing landscape of FTLD-TDP in its different pathological and genetic subtypes. We conducted the largest differential splicing analysis (DSA) in a bulk brain RNAseq dataset for FTLD-TDP, identifying distinct splicing patterns in each subtype. In addition, we uncovered candidate splicing events that may serve as fluid biomarkers for TDP-43 proteinopathies and provide mechanistic insights into FTLD-TDP.

## Materials and methods

### Short-read RNA sequencing

Bulk brain short-read RNA sequencing data from the frontal cortex (FCX) of 149 individuals were analyzed in this study. Brain samples were selected from the Mayo Clinic Florida Brain Bank. We included 27 FTLD-TDP type A, 20 FTLD-TDP type B, 22 FTLD-TDP type C, 24 *GRN* mutation carriers, 34 *C9orf72* repeat expansion carriers and 22 neuropathologically normal individuals, hereafter referred to as controls. Demographic and pathological data of the included individuals is described in Table S1. Available pathological information includes Braak stage, Thal phase, and presence of Lewy bodies.

A detailed description of the data generation was described before [18, 59]. In brief, FCX tissue was collected from the middle frontal gyrus at the level of the nucleus accumbens. RNA was extracted with the RNeasy Mini kit (Qiagen) and quality control was assessed with the 2100 Bioanalyzer and the RNA Nano Chip (Agilent Technologies). Only samples with an RNA integrity number (RIN) ≥ 7 were included. Library preparation was performed using Illumina TruSeq mRNA v2 prep and sequenced at 10 samples/lane as paired-end 101 base pair reads on the HiSeq4000 (Illumina, San Diego, CA). Raw sequencing reads were aligned to the human reference genome (GRCh38) with Spliced Transcripts Alignment to a Reference (STAR; v2.5.2b) [19]. Library quality assessment was performed using the R package *RSeQC* [73].

### Differential splicing analysis (DSA)

DSA was carried out using Leafcutter [36]. In this analysis, splice junctions from STAR aligner were extracted with a minimum of 8 bp as an anchor on each side of the junctions and a maximum intron size of 500 kb using Regtools [16]. Then the introns were clustered with 50 split reads supporting each junction. A Dirichlet-Multinomial generalized linear model was used to identify intron excision events, adjusting for the covariates age at death, sex, and RIN. The annotation of the intron junctions was performed using the GENCODE v42 reference transcriptome.

Two criteria were established to consider an intronic junction within a cluster as differentially spliced: (i) it had to be located within a statistically significant cluster (false discovery rate (FDR) < 0.05) and (ii) it should exhibit a difference of 10% or more between groups (│Δ percent selected index (PSI)│ > 0.1). Significant clusters were classified as cassette exons, using leafviz, if they contained 3 introns with 2 child introns and 1 parent intron. The directionality was calculated based on the effect size of the introns. A skipped cassette exon would have a positive effect size for the parent intron and negative effect sizes for the two child introns, whereas an included exon would have the reverse. Splicing events were visualized using leafviz. Events with │ΔPSI│ < 0.01 were excluded from the figures.

Over-representation analyses (ORA) were performed using the package *clusterProfiler* in R (v4.2.3) [77] and the Biological Process ontology from the Gene Ontology (GO) knowledgebase [3]. Pathways were considered significant if they reached the threshold of FDR < 0.05. All the genes with identified splicing clusters were used as background. The results of the analysis were visualized as a network using the R package *enrichplot*, showing the 20 most significant pathways [79]. The similarity of the terms was calculated with the Jaccard correlation coefficient. Enrichment clusters were calculated using the k-means method, and clusters with less than 3 connected nodes were not shown. Protein–protein interaction networks were built using STRING [67], and non-connected nodes were excluded from the network.

### Cell-type proportion estimation and DSA adjustment

To estimate the cell-type proportions of the five most abundant cell populations (neurons, oligodendrocytes, astrocytes, microglia and endothelial cells) in our bulk brain short-read RNAseq data, we first counted how many aligned sequencing reads were mapped to each feature with HTSeq [5]. Then, cell proportions were calculated with the R package *DSA* [81] using specific cell markers for each cell type from the R package *BRETIGEA* [45], as we described before [59]. Differences in cell-type proportions were assessed using Kruskal–Wallis rank-sum test, and pairwise comparisons were assessed with a Wilcoxon rank-sum test. P values were corrected by multiple comparisons using Bonferroni’s correction.

To adjust our DSA for the differences in the cell-type proportions between FTLD-TDP patients and controls, we first performed a principal component analysis (PCA) with the estimated proportions of the five cell types. The first three principal components (PCs), accounting for more than 80% of the variance, were added as covariates in Leafcutter (together with the above-mentioned age at death, sex and RIN) to perform a DSA adjusting for the differences in the cell-type proportions.

### Identification of TDP-43 binding sites

The identification of TDP-43 binding sites was carried out using the public database CLIPdb, which is a manually curated database of publicly available cross-linked immunoprecipitation (CLIP) data embedded within POSTAR3 [80] [http://postar.ncrnalab.org]. We looked specifically into those annotated TDP-43 binding sites that have been identified in the human brain (using protocols iCLIP-CIMS and iCLIP-Piranha_0.01). A binding site was considered ‘close’ to a splicing event if it was within 500 base pairs of the splicing event.

### Generation and culture of human iPSC-derived neurons

The human iPSC line was obtained from the European Bank for induced pluripotent Stem Cells (https://ebisc.org/WTSIi040-A). Differentiation of human iPSCs into cortical glutamatergic projection neurons was adapted from a previously established protocol [64]. iPSCs were seeded on Matrigel (Corning)-coated six-well plates (Corning) in mTeSR1 medium (Stemcell Technologies) supplemented with 10 µM Y-27632 dihydrochloride (ROCKi; Stemcell Technologies) and maintained in mTeSR1 medium. Neural induction was carried out in highly confluent iPSC cultures (> 80% confluency) using dual SMAD inhibition (10 µM SB431542 and 1 µM LDN193189) in neuronal maturation medium (NMM), which consists of a 1:1 mixture of N-2 and B-27-containing media. N-2 medium is composed of DMEM/F-12 GlutaMAX, 1X N-2, 5 µg/ml insulin, 1X non-essential amino acids, 100 µM 2-mercaptoethanol and 1 mM sodium pyruvate. B-27 medium consists of Neurobasal, 1X B-27, 1X Glutamax, 100 U/mL penicillin–streptomycin). After the formation of a neuroepithelial sheet, cells were passaged with Dispase II (Sigma) at days in vitro (DIV) 10, as small clusters onto wells Matrigel-coated six-well plates. Upon passaging at DIV10, differentiating cells were allowed to form neural rosettes in the presence of 20 ng/ml bFGF (Bio-Techne) for 4 consecutive days and 1X Revitacell (Life technologies) for 1 day. Cells were further passaged with Dispase II at DIV17 and DIV22 and then frozen as cortical neural progenitor cell (NPC) stocks around day 25 or expanded to obtain a bigger pool of NPCs before freezing. For final plating of NPCs to become mature cortical neurons, cells were dissociated with Accutase (Sigma) and seeded on six-well plates coated with Poly-L-ornithine solution (PLO, Sigma) diluted 1:1000 in 1 × DPBS overnight followed by 5 µg/mL of human laminin (rhLaminin-521; StemCell Technologies) at a density of 750,000 cells/well in NMM medium supplemented with 1X RevitaCell for 1 day. Cells were maintained for > 100 DIV (or as desired) in NMM with half medium changes twice a week, except for the first week after plating, during which the medium was not changed.

### Silencing of *TARDBP* in iPSC-derived neurons

Human iPSC-derived neurons at DIV126 (multiplicity of infection (moi) of ~ 5) were transduced for 48 h with three different lentiviral vectors (Vector Builder) with a short hairpin RNA (LV-shRNA) targeting the *TARDBP* gene and a scramble control (four conditions in total, two replicates each). Each of these shTARDBP had different predicted knock-down (KD) scores and targeted different regions of the gene (shRNA#1: target sequence AGATCTTAAGACTGGTCATT, KD score 10.8; shRNA#2: target sequence GCAATAGACAGTTAGAAAGAA, KD score 5.6; shRNA#3: target sequence GCTCTAATTCTGGTGCAGCAA, KD score 2.6). After 48 h of treatment, 1 mL of NMM medium was added to the cells. On day 5 of treatment, cells were dissociated with Accutase (Sigma) and Actinomycin D, pelleted and frozen.

RNA was extracted from the pellets using the RNeasy Mini kit (Qiagen), quality control was performed with 5300 Fragment Analyzer (Agilent) and the concentration was measured using the Qubit RNA Broad Range Assay Kit (ThermoFisher Scientific) and a Qubit Fluorometer. Reverse transcription was performed using the iScript™ cDNA Synthesis Kit (BioRad). The expression of *TARDBP* was measured by means of qPCR to check the efficiency of the KD using the *Power* SYBR™ Green PCR Master Mix (Applied Biosystems) on a QuantStudio 6 Flex Real-Time PCR system (Applied Biosystems). The following primer sequences were used (Integrated DNA Technologies, IDT): *TARDBP* forward primer (FW) 5′-AATTCTGCATGCCCCAGA-3′, reverse primer (RV) 5′-GAAGCATCTGTCTCATCCATTTT-3′; *RPLP0* (housekeeping gene) FW 5′-TCTACAACCCTGAAGTGCTTGAT-3′, RV 5′-CAATCTGCAGACAGACACTGG-3′; *UBE2D2* (housekeeping gene) FW 5′-CAGTCCCTATCAGGGTGGAGT-3′, RV 5′- AAGGGGTAATCTGTTGGGAAATG-3′. Quantification was performed using the ΔΔC_t_ method [40].

### Long-read cDNA sequencing

Total RNA extraction from brain or iPSC-derived neurons was carried out using the RNeasy Mini kit (Qiagen) and quality control was performed with the 5300 Fragment Analyzer (Agilent). Only samples with a RIN (RNA Integrity Number) ≥ 6 were included. RNA concentration was quantified using the Qubit RNA Broad Range Assay Kit (ThermoFisher Scientific) and a Qubit Fluorometer. Following RNA extraction, a polyA enrichment step was performed using the Poly(A) RNA Selection Kit (V1.5, Lexogen). Subsequent quality control and quantification steps were carried out on mRNA, employing the same methods as described previously for total RNA.

cDNA library preparations were carried out according to the manufacturer’s instructions using the Direct cDNA Sequencing kit (SQK-DCS109) from Oxford Nanopore Technologies (ONT). Libraries were barcoded using the Native Barcoding Expansion 1–12 (PCR-free) kit (EXP-NBD104) and 13–14 kit (EXP-NBD114) from ONT. These barcoded libraries were then loaded onto 11 flow cells (R9.4.1) for brain and 4 flow cells for iPSC-derived neurons and sequenced using the PromethION24 sequencer (ONT). Base calling was performed using the ONT data processing toolkit guppy (v3.4.5). The resulting reads underwent alignment to the human reference genome (hg38, GENCODE v42) using minimap2 [35] in the splice mode. For downstream analyses, IsoQuant (v3.3.0) [60] was used for transcript identification and quantification, using the model_construction_strategy sensitive_ont, as implemented in the GenomeComb pipeline (v0.108.0) [https://github.com/derijkp/genomecomb] using the iso_joint option. After exclusion of novel genes, expression values (gene counts) at gene and transcript level were normalized using *DESeq2* [41].

### In silico peptide prediction of candidate genes

For the 16 genes where candidate splicing events could be detected in the bulk long-read RNA sequencing data, the complementary DNA (cDNA) sequences spanning the exons of these transcripts were extracted using the R Bioconductor packages *Biostrings* [56]and *BSgenome.Hsapiens.UCSC.hg38* [68]. Subsequently, the extracted cDNA sequences were subjected to translation using ORFinder, followed by verification of the predicted proteins via BLASTp analysis [15]. For genes where novel transcripts with the splicing events were not detected in the long-read sequencing data, we initially extracted the cDNA sequence of the MANE selected transcript. Next, we adjusted its sequence based on the specific nature of the splicing event, with the help of the Integrative Genomics Viewer (IGV) [69]: (i) for exon inclusion events, we added the nucleotides corresponding to the cryptic exon (extracting it with *Biostrings*); (ii) for exon skipping events, we excised the entire sequence of the skipped exon; (iii) for alternative 5' or 3' splicing site events, we modified the sequence to accommodate the new position of the splicing site. Following this, we ensured that the region of interest (along with the flanking exons) remained consistent across all gene transcripts. Finally, we translated the modified sequence using ORFinder and verified it with BLASTp.

### Comparison of cryptic splicing events across multiple TDP-43 splicing studies

Two sets of cryptic splicing events ascribed to TDP-43 loss of function from previously published studies were selected to be compared with the ones obtained in the present study. The two selected studies performed DSAs in short-read RNAseq data of two different models of TDP-43 proteinopathy: (i) Seddighi et al. (2024) [63]—This study analyzed TDP-43 KD iPSC-derived neurons using short-read RNA sequencing and MAJIQ for DSA. Cryptic splicing events were defined as those with a false discovery rate (FDR) < 0.05, a ΔPSI > 0.1 and a controls PSI < 0.1; (ii) Ma et al. (2022) [42]—This study investigated TDP-43 negative neuronal nuclei from FTLD-TDP patients using short-read RNA sequencing and both MAJIQ and Leafcutter for DSA. Cryptic splicing events were considered those with an FDR < 0.05 and a ΔPSI > 0.1 in the MAJIQ analysis, and an FDR < 0.05 in the Leafcutter analysis. Our own DSA was performed on short-read bulk brain RNA sequencing data from FTLD-TDP patients and controls. We identified cryptic splicing events as those not annotated in the GENCODE v42 reference transcriptome with an FDR < 0.05 and a ΔPSI > 0.

To compare these lists, we first checked the overlapping genes between pairs of studies and then we compared the coordinates of differential splicing events in each gene to identify the common ones. We defined “perfect matches” as genes exhibiting identical splicing event coordinates across studies. A “partial match” was assigned if only one coordinate position aligned between studies. In the case of the Ma et al. study, a “perfect match” was also declared if both coordinate positions of a given event were present in the other two datasets, since in this study, the given coordinates do not represent each intronic junction (like in our study and in Seddighi et al.) but the two ends of the whole splicing event.

### DSA on publicly available datasets from AMP-AD

From the AMP-AD program, bulk short-read RNAseq data from three studies were obtained and analyzed for differential splicing: (i) the MayoRNAseq study (N = 146), with data from the temporal cortex (TCX) [4]; (ii) the Mount Sinai Brain Bank (MSBB) study, with four different datasets from four Broadmann Areas – BA10 (N = 132), BA22 (N = 120), BA36 (N = 105), and BA44 (N = 113) [74]; and (iii) the Religious Orders Study and Memory and Aging Project (ROSMAP) study (N = 218), with data from the dorsolateral prefrontal cortex (DLPFC) [10, 49]. For each dataset, we conducted harmonized (as previously described [72]) case–control comparisons between pathologically confirmed Alzheimer’s disease (AD) patients and controls. In the ROSMAP study, AD patients were defined by a CogDx score of 4, a Braak score ≥ 4, and a CERAD score ≤ 2, whereas controls had a CogDx score of 1, a Braak score ≤ 3, and a CERAD score ≥ 3. In the MSBB Study, AD cases were classified as having a CDR ≥ 1, a Braak score ≥ 4, and CERAD ≥ 2, while controls were defined as having a CDR ≤ 0.5, a Braak score ≤ 3, and CERAD ≤ 1. In the MayoRNAseq study, direct reported diagnosis was used for classification, based on Braak score ≥ 4 and CERAD > 1 for AD patients and Braak score ≤ 3 and CERAD < 2 for the controls. The demographic and pathological data for the three study cohorts are provided in Table S2 for ROSMAP study, S3 for MayoRNAseq study, and S4 for MSBB study. Available pathological information includes Braak stage and CERAD score for ROSMAP and MSBB, and Braak stage and Thal phase for MayoRNAseq.

DSA were performed for each dataset using Leafcutter [28] and RegTools [9], with parameters consistent with our other DSA analyses. Intronic junctions were annotated using the GENCODE v24 reference transcriptome. The analyses were adjusted for RIN, age at death, sex, and sequencing batch. Only samples with RIN > 5 were included.

### Statistical analysis and figures

Statistical analyses were conducted with the R software (v4.2.3) (not including DSA and ORA). The normality of the variables was assessed graphically (histogram, Q-Q plot) and with Kolmogorov–Smirnov test. For normally distributed variables, unpaired Student’s *t* test, ANOVA and Pearson’s coefficient were used for comparisons between two groups, multiple groups and correlations, respectively. On the other hand, if the variables were not normally distributed, we used the Mann–Whitney U test, Kruskal–Wallis test, and Spearman’s Rho. When applicable, we adjusted the *P* values of post hoc comparisons for multiple comparisons using Bonferroni’s correction. Categorical variables were compared using χ^2^ test. Normal variables are represented as mean (± standard deviation (SD)) and non-normal variables are represented as median (interquartile range (IQR)). To create the figures, we used the R packages *ggplot2* [75], *ggpubr* [32], *pheatmaps* [34], *enrichplot* [79] and *leafviz* [25, 79], together with Adobe Illustrator 2024 (Adobe Systems) and BioRender.

## Results

### DSA in FTLD-TDP brains identifies known and novel splicing alterations

**Fig. 1:**
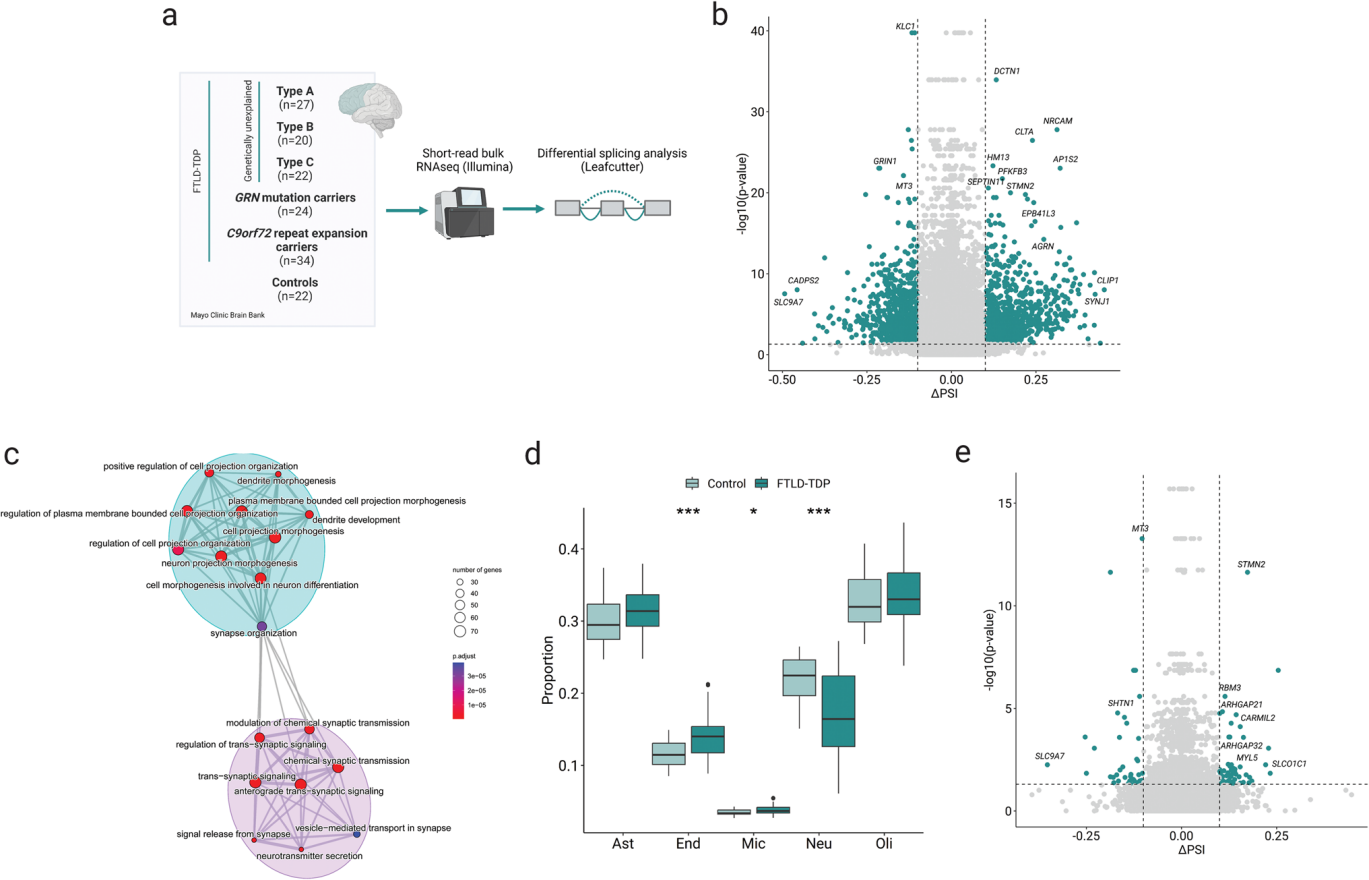
Brain splicing alterations landscape in FTLD-TDP. **a** Workflow of the differential splicing analysis in bulk short-read sequencing data. Figure created with BioRender.com. **b** Volcano plot of the differentially spliced events in FTLD-TDP vs controls without adjusting by cell type proportions. In green, events within a significant cluster (FDR < 0.05) and a│Δ PSI│ > 0.1. **c**. Network of pathways enriched in differentially spliced genes, with the blue module representing pathways involved in dendrite and cell projections, and the pink module with pathways involved in synapse dysfunction. **d** Relative proportions of the major cell types in the brains of FTLD-TDP and controls. Mann–Whitney U test. *P < 0.05, **P < 0.01, ***P < 0.001, ****P < 0.0001. **e** Volcano plot of the differentially spliced events in FTLD-TDP vs controls after adjusting by cell-type proportions. Ast: astrocytes, Mic: microglia, Oli: oligodendrocytes, Neu: neurons, End: endothelial cells

To identify differential splicing events shared by all genetic and pathological FTLD-TDP subtypes, we analyzed bulk RNAseq data from the FCX of the brain in our full cohort using Leafcutter (N = 127/22 FTLD-TDP/controls) (Fig. 1a). Splicing events (intronic junctions) were considered as differentially spliced if they were located within a statistically significant cluster (FDR < 0.05) and exhibited a difference of 10% or more between groups (│Δ PSI│ > 0.1). A cluster is defined as a set of overlapping spliced events.

We identified a total of 1818 differentially spliced events distributed across 1054 clusters (Fig. 1b, Table S5). These clusters belong to 842 unique genes, indicating that, in some genes, there are two or more significant clusters, and that some of the events were in intergenic regions. Among the 1054 clusters, 381 events were categorized as cassette exons, where an intervening exon between two other exons in the mature mRNA sequence was either included or skipped. The majority of these cassette exons were skipped (77%), 18% were included, and the remaining 5% were classified as complex. Of the 1818 differentially spliced events, 69 were categorized as novel, with a novel acceptor (*n* = 26), a novel donor (*n* = 26) or a novel donor–acceptor combination (*n* = 17).

To elucidate the biological pathways affected by these splicing alterations, we conducted an ORA that showed enrichment in two distinct modules (Table S6). The first module included pathways related to synaptic signaling, featuring terms such as “vesicle-mediated transport in synapse” (FDR = 3.68E-05, GO:0099003), “signal release from synapse” (FDR = 2.56E-06, GO:0099643), and “modulation of chemical synaptic transmission” (FDR = 2.53E-06, GO:0050804). Relevant genes associated with these pathways, identified in the top 20 most dysregulated clusters, included *GRIN1* (FDR = 3.84E-26) (Fig. S1a), with an alternative 5' splice site in the final exon of the gene (exon 20) (chr9:137,163,904–137167774, ΔPSI = -0.11), and *SYNJ1*, where the splicing alteration involved the skipping of exons 26, 27, and 28 (chr21:32,641,966–32,645,646, ΔPSI = 0.24, FDR = 3.56E-17) (Fig. S1b).

The second module was linked to pathways involving dendrite and cell projection, including terms such as “dendrite development” (FDR = 1.15E-06, GO:0016358), “regulation of cell projection organization” (FDR = 9.07E-06, GO:0031344), and “neuron projection morphogenesis” (FDR = 1.17E-06, GO:0048812) (Fig. 1c). Genes playing pivotal roles in these pathways and also found among the top 20 most differentially spliced clusters were *NRCAM,* where we observed a higher relative abundance of the junction chr7:108,160,492–108176430 (ΔPSI = 0.31, FDR = 2.68E-32) (Fig. S1c) corresponding to the skipping of exons 28 to 30; *STMN2*, where we detected the cryptic inclusion of an exon between exons 1 and 2 (chr8:79,611,214–79,616,822, ΔPSI = 0.21, FDR = 1.40E-23) (Fig. S1d); *EPB41L3*, which displayed skipping of exon 17 (chr18:5,397,426–5,406,777, ΔPSI = 0.24, FDR = 1.65E-22) (Fig. S1e); and *MT3* (FDR = 4.36E-26) (Fig. S1f), comprising a complex splicing event with several genes from the metallothioneins family involved.

It is widely acknowledged that bulk RNASeq analysis inherently lacks the ability to discern expression or splicing changes occurring in distinct cell populations. To surpass this limitation, we performed a relative cell-type proportion estimation based on the expression of known cell-type markers. As expected, we observed a distinct pattern of cellular composition in FTLD-TDP patients compared to controls, with a significantly lower relative proportion of neurons in the FTLD-TDP group [0.16 (± 0.09) vs. 0.22 (± 0.05) respectively, adj. *P* value = 0.0002]. The relative proportion of other cell-type populations were elevated in FTLD-TDP as compared to controls, with endothelial cells and microglia exhibiting a statistically significant increase [0.14 (± 0.036) vs. 0.11 (± 0.03), adj. *P* value = 0.0005 for endothelial cells; 0.037 (± 0.008) vs. 0.033 (± 0.006), adj. *P* value = 0.01 for microglia] (Fig. 1d). To account for these differences in our DSA, we performed a PCA with the estimation of the proportion of all the studied cell types and then added the first three PCs as covariates in the DSA. These three PCs together accounted for more than 80% of the total variance of the data (PC1 50.6%, PC2 27.7%, PC3 11.4%) and explained almost completely the variance of each cell type. The cell-type-adjusted DSA led to a notable reduction in the number of significant splicing events (Fig. 1e). Specifically, 96 events distributed across 67 clusters were identified as significant when applying the selected thresholds, corresponding to 59 unique genes (Table S7). Importantly, *SYNJ1*, *MT3*, and *STMN2* maintained significant even after adjustment, suggesting their involvement in FTLD-TDP is at least in part independent from specific cell loss.

### FTLD-TDP subtypes exhibit distinct patterns of splicing alterations

**Fig. 2:**
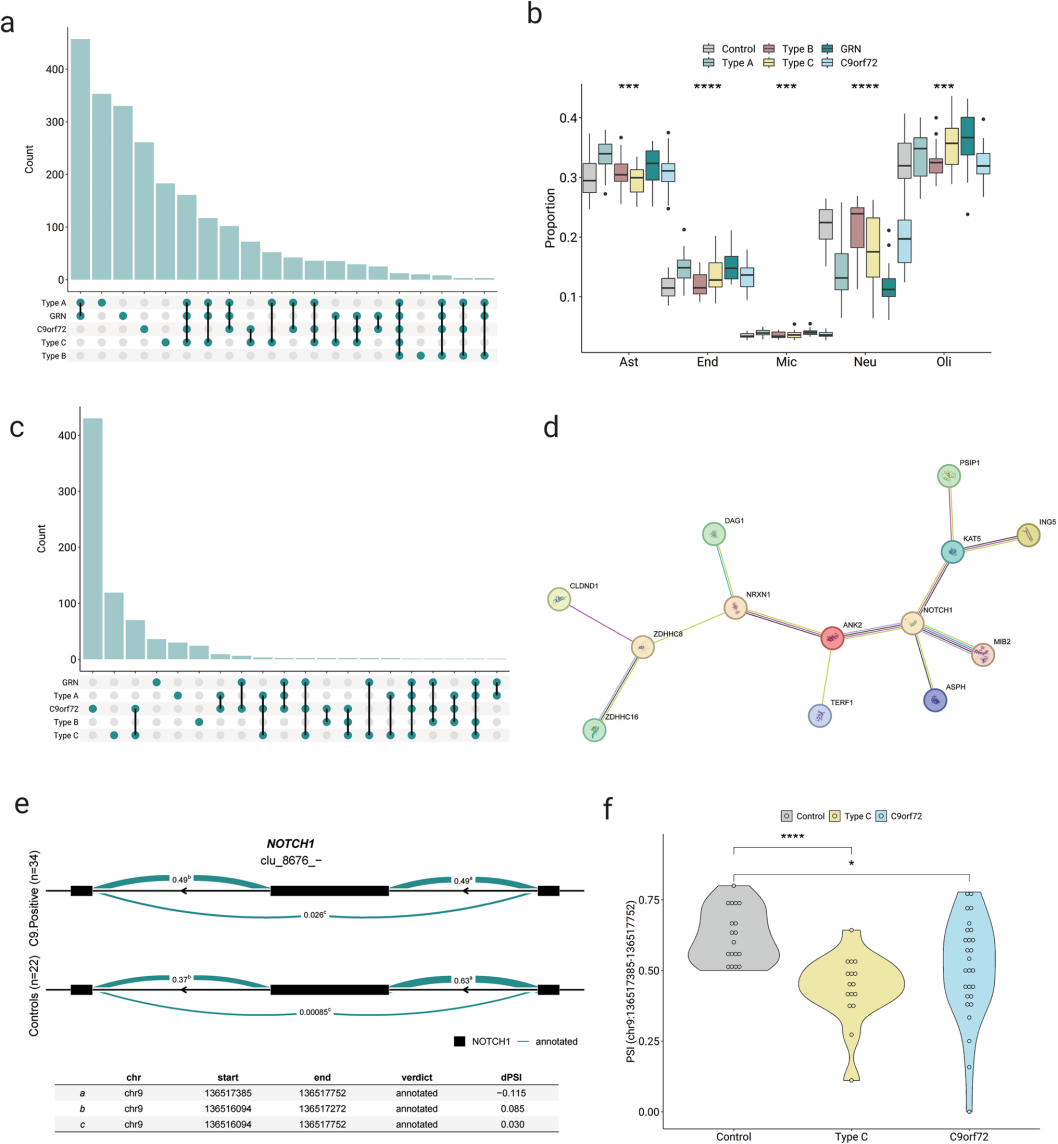
FTLD-TDP subtypes show distinct splicing profiles. **a** Upset plot of the differentially spliced clusters (FDR < 0.05) among FTLD-TDP subtypes, without adjusting by differences in cell proportions. **b** Relative proportions of the major cell types in the FTLD-TDP subtypes and controls. Mann–Whitney U test, adjusted by Bonferroni. **c** Upset plot of the differentially spliced clusters (FDR < 0.05) among FTLD-TDP subtypes, adjusting by differences in cell proportions. **d** Protein–protein interaction network between connected genes that are commonly spliced in *C9orf72* repeat expansion carriers and FTLD-TDP type C. **e.** Sashimi plot for *NOTCH1* splicing cluster. The numbers represent the PSI of that splice junction **f.** Violin plot of the PSI of the splicing event in *NOTCH1* in controls, *C9orf72* repeat expansion carriers and FTLD-TDP type C. Ast: astrocytes, Mic: microglia, Oli: oligodendrocytes, Neu: neurons, End: endothelial cells. *P < 0.05, **P < 0.01, ***P < 0.001, ****P < 0.0001

Next, we inspected if different FTLD-TDP pathological subtypes and *GRN* and *C9orf72* mutation carriers had distinct splicing profiles in the FCX. DSA without adjusting for cell-type proportions, comparing each of the disease subgroups to controls, revealed that FTLD-TDP type A exhibited the highest number of identified differentially spliced clusters (*N* = 1352) (Table 1, Table S8), followed by *GRN* mutation carriers (*N* = 1281) (Table S9), *C9orf72* repeat expansion carriers (*N* = 754) (Table S10), and FTLD-TDP type C (*N* = 703) (Table S11). In contrast, individuals with FTLD-TDP type B showed notably fewer differentially spliced clusters (*N* = 77) compared to the other pathological subtypes and mutation carriers (Table S12). Twelve clusters were found to be differentially spliced in all groups, and one hundred sixty-one were common in all groups except type B. The significant clusters in all groups were in the following unique genes: *NRCAM, TPD52L2, PDE4DIP, SLC25A2, CADPS2, ATP1B3, PPFIA1, PLEC, ASPH, ZRANB1, FAM200B* and one in an intergenic region. Importantly, the cluster that leads to a cryptic exon in *STMN2* was found differentially spliced in all groups except for type C, although it was still significant (FDR = 1.06E-08) in this subtype but the ΔPSI associated with the novel event was 0.06 (below the threshold set at 0.1 in our analysis). Furthermore, FTLD-TDP type A and *GRN* mutation carriers shared the highest number of unique differentially spliced clusters (*N* = 457), clearly distinct from the other groups (Fig. 2a).

**Table 1:** Counts of differentially spliced events, clusters, and genes in FTLD-TDP brains vs controls

	Events	Clusters	Unique genes
Unadjusted by cell proportion			
FTLD-TDP (all)	1818	1054	842
FTLD-TDP type A	2480	1352	1074
FTLD-TDP type B	77	44	38
FTLD-TDP type C	1176	703	637
*GRN* mutation carriers	2444	1281	1051
*C9orf72* repeat expansion carriers	1214	754	685
Adjusted by cell proportion			
FTLD-TDP (all)	96	67	59
FTLD-TDP type A	83	49	43
FTLD-TDP type B	71	31	25
FTLD-TDP type C	322	202	191
*GRN* mutation carriers	105	51	48
*C9orf72* repeat expansion carriers	810	529	499

The included clusters have a false discovery rate (FDR) < 0.05, and the events have a | Δ percent spliced in index (PSI) | > 0.1

Focusing on the enriched biological pathways within each subgroup, we observed the presence of the “dendrite and cell projection” module, consistent with the analysis in all FTLD-TDP samples, in all subgroups except for type B. Notably, in type B, no significant enrichment of biological pathways was identified, likely attributed to the limited number of splicing events in this subgroup. However, in each of the other subgroups, a distinct module was identified in addition to the common one. In FTLD-TDP type A, a module of pathways related to synaptic signaling was identified, shared also with the analysis with all FTLD-TDP individuals (Fig. S2a, Table S13). In FTLD-TDP type C, the additional module was related to the establishment of spindle orientation and organization, with features like “establishment of mitotic spindle localization” (FDR = 0.0031, GO:0040001), “establishment of spindle orientation” (FDR = 0.019, GO:0051294), and “establishment of spindle localization” (FDR = 0.0231, GO:0051293) (Fig. S2b, Table S14). Examining the mutation carriers’ subgroups revealed that *GRN* mutation carriers exhibited enrichment of pathways in two additional modules: regulation of small GTPase activity, encompassing “small GTPase mediated signal transduction” (FDR = 1.77E-05, GO:0007264), “regulation of small GTPase mediated signal transduction” (FDR = 7.23E-05, GO:0051056) and “regulation of GTPase activity” (FDR = 0.0001, GO:0043087), and pathways related to synaptic signaling, shared with FTLD-TDP type A and all FTLD-TDP (Fig. S2c, Table S15). Finally, in *C9orf72* repeat expansion carriers, the unique module identified encompassed pathways related to the regulation of mRNA splicing, including terms as “regulation of mRNA splicing, via spliceosome” (FDR = 0.0299, GO:0048024), “regulation of mRNA processing” (FDR = 0.03, GO:0050684), and “mRNA splice site selection” (FDR = 0.0382, GO:0006376) (Fig. S2d, Table S16).

We also estimated the proportions of the five most abundant cell types in the brain within each FTLD-TDP subgroup. FTLD-TDP type A and *GRN* mutation carriers exhibited a similar cellular composition, showing a significant reduction in neurons compared to controls (see Fig. 2b) (Type A vs controls adj. *P* value < 0.0001; *GRN* mutation carriers vs controls adj. *P* value < 0.0001) but also to other groups (Table S17). After accounting for the major cell-types proportions in our analyses, a noticeable reduction in the number of differentially spliced clusters was observed in FTLD-TDP type A and *GRN* mutation carriers *N* = 49 and 51, respectively (Tables S18 and S19), with only one cluster differentially spliced in both subgroups. This suggests that most splicing alterations in these two groups can be attributed to their unique and distinct cellular compositions (Fig. 2c). Furthermore, after cell-type adjustments, *C9orf72* repeat expansion carriers appeared to be most affected by aberrant splicing (N = 529) (Table S20), followed by FTLD-TDP type C patients (N = 202) (Table S21). Interestingly, 78 differentially spliced clusters were shared between FTLD-TDP type C and *C9orf72* repeat expansion carriers, potentially signifying common underlying disease processes in these two groups. Inspection of the 78 shared clusters identified *NOTCH1* as a node with 4 interacting partners (*KAT5*, *MIB2*, *ASPH*, and *ANK2*) that also exhibit aberrant splicing, suggesting potential disruptions in the Notch signaling pathway (Fig. 2d). The most differentially spliced event identified in *NOTCH1* (Fig. 2e) corresponded to the intron connecting exons 8 and 9 of the canonical transcript (ENST00000651671.1) of the gene, with the coordinates chr9:136,517,385–136,517,752. This event had a ΔPSI = − 0.115, corresponding to a higher presence in the controls (Fig. 2f).

### Identification of novel cryptic splicing alterations in FTLD-TDP

We next focused on identifying cryptic splicing events that could contribute to FTLD-TDP disease mechanisms or serve as biomarkers for TDP-43 pathology. We first searched in our data on the full patient cohort for those events that were novel (not annotated in the GENCODE v42 transcriptome), exhibited a difference of more than 10% between patients and controls, and were elevated in FTLD-TDP as compared to controls. This resulted in the identification of 30 cryptic events in 30 unique genes.

Clustering analysis and the heatmap of our 149 samples (Fig. S3a), focusing on the 30 cryptic splicing alterations, revealed that the control samples predominantly clustered together, with only one exception, indicating a consistent pattern among them. Importantly, certain FTLD-TDP patients were also part of this clade, exhibiting minimal cryptic splicing. We observed that these control-like patients have a higher neuronal proportion [0.234 (0.199–0.259) vs 0.135 (0.114–0.173), *P* < 0.0001] (Fig. S3b), suggesting less neurodegeneration and likely less TDP-43 pathology in the FCX of these patients. Most of the patients clustering with the controls were FTLD-TDP type B, with 70% of these patients grouping with the controls. In contrast, the proportion of patients grouping with the controls was lower in other groups: 25% for type A, 28% for type C, 12% for *GRN* mutation carriers, and 38% for *C9orf72* repeat expansion carriers (χ^2^ *p* = 0.001) (Fig. S3c).

Among the 30 genes harboring cryptic splicing alterations, 24 were protein-coding, with only 2 of them being reported in previous studies (*STMN2* and *ARHGAP32*), 4 were pseudogenes, 1 was a miRNA, and 1 was a long non-coding RNA (Table 2). For the protein-coding genes, we used in silico predictions to determine the potential cryptic peptides resulting from these splicing events (Table 2). We also investigated the cell specificity of the 24 protein-coding genes harboring the cryptic events making use of the publicly available data from the Human Protein Atlas. We observed that cryptic events were not only expressed by neurons but also by other glial cell types. In fact, six of the genes were mainly expressed in the neuronal cell types, four were enriched in oligodendrocytes, and three genes were mainly expressed in astrocytes. The remaining genes were not found enriched in a specific cell type (Fig. S3d).

**Table 2:** Top 30 significant cryptic splicing events with the highest difference between FTLD-TDP brains and controls

Gene name	Gene type	Event type	Intronic coordinates	ΔPSI	FDR	PSI controls	PSI FTLD-TDP	Reported in literature	Novel transcript confirming the event in long-read brain	Predicted protein consequence
*STMN2*	Protein coding	EI	chr8:79,611,214–79,616,822	0.218	1.55E-20	0.0004	0.219	Yes	Yes	Trunc. proteoform
*DLG3*	Protein coding	ES	chrX:70,493,475–70498520	0.125	1.96E-10	0.0395	0.165	No	No	Sequence missing
*PPP1R3F*	Protein coding	ES	chrX:49,282,063–49299276	0.145	5.33E-08	0.1039	0.245	No	Yes	Alt. C-terminal
*PLCH1*	Protein coding	A5SS	chr3:155,483,048–155485356	0.234	3.33E-07	0.0290	0.264	No	No	No change
*PLEKHA6*	Protein coding	A3SS	chr1:204,274,809–204350880	0.121	7.61E-06	0.2939	0.416	No	No	Alt. N-terminal
*FAM200B*	Protein coding	A5SS	chr4:15,686,462–15,686,928	0.100	1.52E-05	0.0606	0.161	No	Yes	No change
*PTP4A3*	Protein coding	A3SS	chr8:141,418,018–141421388	0.221	1.59E-05	0.210	0.431	No	Yes	No change
*MYL5*	Protein coding	EI	chr4:681,321–681,893	0.232	1.78E-05	0.0743	0.306	No	Yes	Trunc. proteoform
*GRIP2*	Protein coding	ES	chr3:14,517,213–14,520,110	0.291	3.92E-05	0.0341	0.325	No	No	Sequence missing
*FARSB*	Protein coding	EI	chr2:222,643,005–222646801	0.132	5.52E-05	0.081	0.213	No	Yes	Trunc. proteoform
*DOCK3*	Protein coding	ES	chr3:51,330,223–51333158	0.266	0.000158	0.1474	0.413	No	No	Sequence missing
*ARHGAP32*	Protein coding	EI	chr11:128,992,046–128998319	0.236	0.000207	0.0647	0.301	Yes	Yes	Trunc. proteoform
*NAT14*	Protein coding	A5SS	chr19:55,485,780–55487054	0.158	0.000347	0.0945	0.252	No	Yes	Alt. C-terminal
*LINC01202*	LncRNA	A5SS	chr5:161,893,763–162,001,120	0.123	0.000636	0.0819	0.204	No	Yes	NA
*GTF2H2B*	Pseudogene	A5SS	chr5:70,401,668–70420249	0.140	0.001115	0.0744	0.214	No	Yes	NA
*CATSPERZ*	Protein coding	ES	chr11:64,300,987–64303773	0.134	0.003234	0.0583	0.193	No	No	Alt. C-terminal
*AUH*	Protein coding	A5SS	chr9:91,294,797–91,296,021	0.124	0.003812	0.1207	0.245	No	No	Alt. C-terminal
*ENSG00000269707*	Pseudogene	A5SS	chr2:104,854,485–104854628	0.110	0.004717	0.1722	0.282	No	No	NA
*PDE4DIPP2*	Pseudogene	A3SS	chr1:120,641,759–120665063	0.188	0.005237	0.0522	0.24	No	No	NA
*NCKAP5L*	Protein coding	A3SS	chr12:49,792,051–49792367	0.138	0.007229	0.2085	0.347	No	Yes	Alt. C-terminal
*SLCO1C1*	Protein coding	A5SS	chr12:20,717,230–20717703	0.192	0.016497	0.3094	0.502	No	Yes	Alt. C-terminal
*MLXIPL*	Protein coding	A3SS	chr7:73,599,695–73,603,395	0.122	0.017979	0.0975	0.22	No	No	Alt. N-terminal
*PYROXD2*	Protein coding	A5SS	chr10:98,385,184–98,387,201	0.126	0.019634	0.0895	0.215	No	No	Alt. C-terminal
*WDR17*	Protein coding	ES	chr4:176,142,069–176148133	0.173	0.019818	0.0693	0.242	No	No	Sequence missing
*TMEM125*	Protein coding	A5SS	chr1:43,272,339–43,273,634	0.119	0.034873	0.1269	0.246	No	Yes	Alt. C-terminal
*CHRM3*	Protein coding	A5SS	chr1:239,632,286–239,634,323	0.130	0.035674	0.0165	0.147	No	Yes	Alt. C-terminal
*SDHAP1*	Protein coding	ES	chr3:195,960,086–195965429	0.116	0.036582	0.0867	0.203	No	No	NA
*MIR762HG*	MicroRNA	A3SS	chr16:30,894,410–30894548	0.111	0.046678	0.0503	0.161	No	No	NA
*ADAM23*	Protein coding	A5SS	chr2:206,550,160–206550623	0.129	0.047029	0.0807	0.21	No	No	Alt. C-terminal
*PCNX2*	Protein coding	A5SS	chr1:233,065,661–233090061	0.1143	0.049119	0.0748	0.190	No	No	Alt. C-terminal

Cryptic events (not annotated) with a Δ Percent Spliced in Index (PSI) > 0.1 are shown. *LncRNA* long non-coding RNA, *EI* exon inclusion, *ES* exon skipping, *A5SS* alternative 5' splice site, A3SS: alternative 3' splice site, *Trunc* truncated, *NA* not applicable, *Alt* alternative, *PSI* percent spliced in index, *FDR* false discovery rate

To gain additional evidence of the existence of these cryptic splicing events, we generated long-read cDNA sequencing in a subset of brain samples (*n* = 16, including 9 FTLD-TDP patients). Among all identified transcripts (256,518 excluding novel genes), we inspected if we could detect transcripts containing the junctions belonging to the cryptic events listed in Table 2. In 16 of the genes of interest, we found one or more transcripts with novel cryptic junctions from Table 2 (28 in total) (Table S23). In most of these genes (11 out of 16), the expression level of the transcripts containing the cryptic event was higher in FTLD-TDP patients, reaching statistical significance for 4 genes despite the small cohort size: *STMN2* [16.6 (6.39–33.4) vs 0 (0–0), *P* = 0.002], *PPP1R3F* [20.4 (19.5–39.7) vs 17.1 (14.1–17.4), *P* = 0.008], ENSG00000269707 [9.58 (5.98–19.2) vs 4.71 (0.47–6.88), *P* = 0.02] and *SLCO1C1* [29.3 (13.5–46.3) vs 8.03 (0.80–11.2), *P* = 0.04]. The identification of transcripts with cryptic splicing events in our in-house long-read sequencing dataset confirms the presence of these novel events but does not exclude the possibility of the existence of the undetected ones. The small cohort of nine FTLD-TDP patients and insufficient coverage for some genes may have hindered their identification.

### Characterization of the relationship between the cryptic splicing events and TDP-43 loss of function

Having identified novel cryptic splicing events in FTLD-TDP brains, we further investigated the relationship between the loss of TDP-43 function in the nucleus and the presence of these cryptic events. We first queried CLIPdb and identified that in 20 of the 30 genes containing cryptic transcripts, at least one TDP-43 binding site was predicted in the human brain, with 7 genes (*DLG3, PPP1R3F, PLCH1, GRIP2, FARSB, ARHGAP32* and *PCNX2*) containing TDP-43 binding sites located either within the coordinates of the cryptic event or within 500 base pairs of one of the adjacent regions (Table S24). We next performed long-read cDNA sequencing on iPSC-derived cortical neurons with a gradient of progressively reduced *TARDBP* expression (Fig. S4a). Transcripts harboring the cryptic events were observed in at least one condition for 13 of the 30 genes (Table S25). However, when correlating the expression of *TARDBP* with the expression of each cryptic transcript, *STMN2* [*r* = − 0.6, *P* = 0.1] (Fig. S4b) and *ARHGAP32* [*r* = − 0.7, *P* = 0.081] (Fig. S4c) were the only transcripts that negatively correlated (defined as *r* < − 0.5) with *TARDBP* expression, even though these correlations did not reach statistical significance, likely due to the small sample size. *ARHGAP32* and *STMN2* were also the only genes where individual CE PSI values from DSA in short-read RNAseq correlated with age of disease onset, reaching significance for *ARHGAP32* [*r* = − 0.28, *P* = 0.002] (Fig. S5a) and near-significance for *STMN2* [*r* = − 0.18, *P* = 0.06] (Fig. S5b). This prompted the question as to whether the majority of identified splicing alterations in FTLD-TDP could be driven by general neurodegeneration rather than TDP-43 loss of function. To test this, we queried publicly available bulk short-read RNAseq datasets from Alzheimer’s disease patients and controls (AMP-AD program). We performed DSA analyses across six different datasets, four of which belong to the MSBB study, that includes four different brain regions (BA10, BA22, BA36 and BA44), and the other two coming from the MayoRNAseq study (TCX) and the ROSMAP study (DLPFC). We found that the majority of the 30 events observed in FTLD-TDP (*n* = 16) were also detected as significant cryptic splicing events in the MayoRNAseq AD study (*DLG3*, *PLCH1*, *PTP4A3*, *PLEKHA6*, *PPP1R3F*, *GRIP2*, *LINC01202, DOCK3*, *SDHAP1*, *MIR762HG*, *NCKAP5L*, *NAT14, AUH, FARSB, FAM200B* and *MYL5*) (Table S26). Remarkably, eight of these events showed substantial differences between AD patients and controls (ΔPSI > 0.1). Most of the remaining events followed the same direction as observed in FTLD-TDP (higher expression in AD compared to controls), except for *LINC01202* which showed a slight negative ΔPSI (ΔPSI = − 0.0012). In addition, two events were also significant in the MSBB BA36 dataset, *FARSB* (ΔPSI = 0.08) and *DOCK3* (ΔPSI = 0.03). Lastly, the cryptic splicing event in *CATSPERZ* was in a significant cluster in the ROSMAP dataset (ΔPSI = 0.06). Interestingly, the CE in *STMN2* was not identified in any of the AD datasets, and the event in *ARHGAP32* was detected at very low levels in the ROSMAP, MayoRNAseq, and MSBB BA36 datasets but without significant differences between AD patients and controls. Together these analyses suggest that splicing alterations observed in *STMN2* and *ARHGAP32* likely resulted from TDP-43 dysfunction, whereas most of the other observed splicing alterations resulted from general neurodegenerative disease processes.

### Comparative analysis with other TDP-43 datasets

**Fig. 3:**
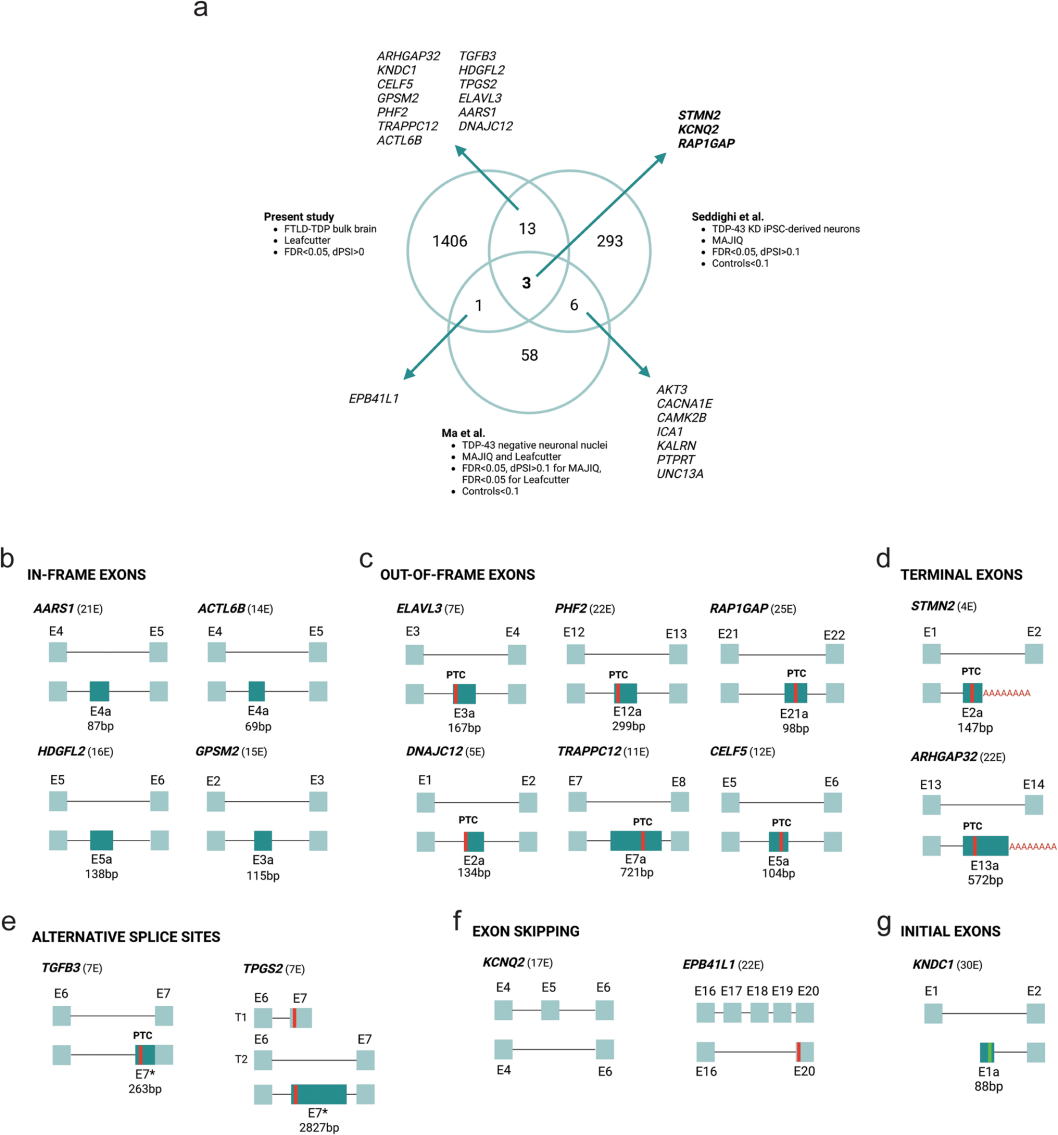
Cryptic splicing in FTLD-TDP. **a** Venn diagram representing genes with perfect splicing matches from the three examined studies. The lower panels represent **b** in-frame exons, **c.** out-of-frame exons, **d** terminal exons, **e** alternative splice sites, **f** exon skipping, and **g** initial exons of those splicing events with perfect matches in the present study and any of the other datasets. For each gene, the upper diagram represents the annotated canonical transcript (MANE selected transcript) and the lower diagram represents the cryptic transcript. Sizes of the cryptic exons are representative to the real size of the exon, but size of the annotated exons are equal in all genes. Next to each gene name, the total number of exons of the canonical transcript is shown. The red lines in the cryptic exons represent premature termination codons (PTC), and the green lines represent novel start codons. The star next to the exon name indicates that part of the exon is the same as the canonical one. Figure created with BioRender.com

We next investigated why, with the exception of *STMN2* and *ARHGAP32*, previously reported cryptic splicing events resulting from TDP-43 loss of function were not identified in our study. We found that known cryptic events, including *ACTL6B*, *HDGFL2*, and *KCNQ2* could be detected in statistically significant clusters in our data when comparing FTLD-TDP to controls (FDR < 0.05); however, the observed differences were very small (ΔPSI *ACTL6B* = 0.01; ΔPSI *KCNQ2* = 0.003; ΔPSI *HDGFL2* = 0.01). In fact, we detected 2996 cryptic splicing events in significant clusters when extending our analysis to those with a positive ΔPSI (ΔPSI > 0) without a specific threshold. To compare this list with cryptic events reported in two previous publications, we first assessed the overlap at the gene level by identifying common genes with cryptic splicing events. This analysis revealed 52 shared genes between Seddighi et al. (TDP-43 KD neurons) [63] and the present study, and 24 between the present study and Ma et al. (TDP-43 negative neuronal nuclei) [42]. Next, at a coordinate level, we identified three splicing events that were considered perfect matches and commonly identified as TDP-43 targets across the three datasets: *STMN2*, *KCNQ2*, and *RAP1GAP*. In addition, 13 splicing events were perfect matches only in TDP-43 KD neurons and the FTLD-TDP bulk brains, e.g., *ARHGAP32, KNDC1, CELF5, GPSM2, PHF2, TRAPPC12, ACTL6B, TGFB3, HDGFL2, TPGS2, ELAVL3, AARS1*, and *DNAJC12*, and one event was uniquely overlapping between the TDP-43 negative neuronal nuclei and the FTLD-TDP bulk brains (*EPB41L1*) (Fig. 3a, Tables S27, S28, and S29). Remarkably, none of the cryptic events consistently observed across studies showed a difference greater than 4% (ΔPSI < 0.04) except for *STMN2* and *ARHGAP32*, which showed a difference in abundance between FTLD-TDP and controls exceeding 20% (ΔPSI > 0.2).

For the 17 splicing events with perfect matches, we performed in silico peptide prediction to study their translatability and biomarker potential. Depending on their peptide product, we classified the splicing events in six different categories: (i) in-frame exons, with *AARS1, ACTL6B, HDGFL2* and *GPSM2* (Fig. 3b); (ii) out-of-frame exons, with *ELAVL3, PHF2, RAP1GAP, DNAJC12, TRAPPC12, CELF5* (Fig. 3c); (iii) terminal exons, with *STMN2* and *ARHGAP32* (Fig. 3d); (iv) alternative splice sites, with *TGFB3* and *TPGS2* (Fig. 3e); (v) exon skipping, with *KCNQ2* (Fig. 3f) and (vi) initial exon, with *KNDC1* (Fig. 3g). The out-of-frame exons all lead to premature stop codons within the novel sequence of the cryptic exon. In silico predicted peptide sequences can be found in Table S30.

In addition, we also identified several partial matches when comparing the three studies. Specifically, we identified nine partial matches (*ANKRD36C, CERS4, LINC00963, MADD, PPFIBP1, RAB40B, RAI2, RNASET2*, and *SEPTIN11*) when comparing our set of splicing events with the ones from Seddighi et al., and five when comparing it with the set identified in Ma et al. (*MADD*, *NFIX, PACRGL, PLEKHA1*, and *UQCRC2*) (Tables S27, S28 and S29).

## Discussion

In this study, we conducted the largest DSA in bulk brain tissues derived from the FCX of FTLD-TDP patients, including genetically unexplained patients characterized by different pathological subtypes and known mutations (*C9orf72* and *GRN*). Although differential splicing and splicing alterations have been widely studied in TDP-43 proteinopathies, this is the first study that explores the splicing patterns across various pathological subtypes and genetic forms of FTLD-TDP.

Our study revealed the uniqueness of the FTLD-TDP subtypes in terms of splicing patterns, in line with recent studies that have highlighted genetic, transcriptomic, and histopathological differences within FTLD-TDP subtypes [6, 7, 17, 58, 59]. The highest number of differential splicing events after cell-type proportion adjustments were observed in *C9orf72* repeat expansion carriers, with most being unique alterations not found in other FTLD-TDP subtypes. The extensive splicing alterations likely result from the specific disease pathology associated with the *C9orf72* repeat expansion. For example, the (GGGGCC)_n_ repeat RNA was recently found to disrupt the nuclear speckle integrity and this, in turn, induces alternative splicing defects [76]. Also, extensive cryptic splicing was recently reported in the cerebellum of *C9orf72* repeat expansion carriers, a brain region susceptible to the accumulation of RNA foci and DPR (dipeptide repeat) proteins in the absence of TDP-43 pathology, emphasizing the contribution of non-TDP-43 pathologies to the splicing alterations [71]. In the latter study, the authors identified RNA splicing regulation as an enriched biological process in their differentially spliced genes when comparing *C9orf72* repeat expansion carriers to controls, similar to what we observed in the FCX. In contrast, we observed the lowest number of alternative splicing alterations in FTLD-TDP type B. We attribute this to the fact that patients with this pathological subtype typically present motor neuron disease symptoms [43], indicating that FCX is probably less affected in type B than in other subtypes, and that other cortical regions like the motor cortex should be investigated for this FTLD-TDP subtype in the future. For the remaining subtypes, FTLD-TDP A and *GRN* carriers were found to share a large number of differential splicing events, in line with our previous observation of a shared gene expression profile in FTLD-TDP A and *GRN* carriers [59]. However, after correcting for cell-type proportions, nearly all these significant events were lost, indicating that the majority of the splicing changes were the result of neuronal loss and other changes in the cellular composition. In contrast, for FTLD-TDP type C, we observed a relatively large number of differentially spliced genes, even after cell-type corrections.

We also observed similarity in terms of splicing patterns, in particular between FTLD-TDP type C and *C9orf72* repeat expansion carriers, which was quite unexpected, since *C9orf72* expansion carriers are typically classified histopathologically as FTLD-TDP type A or type B but not FTLD-TDP C. Moreover, FTLD-TDP C is considered almost exclusively non-familial [52]. The splicing similarities observed between both groups appear to be, in part, linked to Notch signaling pathways, as *NOTCH1* and 4 of its interaction partners (*KAT5, MIB2, ASPH,* and *ANK2*) undergo differential splicing in both groups. Notch1, as key receptor in the Notch signaling pathway, plays a crucial role in stem cell maintenance and differentiation during development, but it is also essential for cell migration and synaptic plasticity in the adult brain [1]. *C9orf72* repeat expansion has already been linked to the Notch signaling pathway, as it has been shown that some dipeptide repeat proteins (DPRs), specifically poly(GR) peptides, can impair this pathway [78]. On a related note, the Notch pathway, including *NOTCH1*, was found over-represented in an analysis of significant sub-genome-wide significant risk genes in clinical svPPA patients [12], which is a clinical subtype enriched in patients with underlying FTLD-TDP type C pathology [66], supporting the relevance of the Notch pathway in this FTLD-TDP subtype. In addition, the latest FTLD-TDP GWAS uncovered risk loci in FTLD-TDP type C with a role in the Notch signaling pathway [58]. The observed splicing alterations in *NOTCH1* and its receptors in FTLD-TDP type C and in *C9orf72* repeat expansion carriers could lead to a disbalance in the Notch signaling pathway, altering key functions in the adult brain and exacerbating neurodegeneration processes. However, we cannot exclude that there might be a neurodevelopmental component too. In fact, the *C9orf72* repeat expansion has been reported to affect neurodevelopment via alteration of neural stem cell maintenance, which in turn reduces the thalamic volume and cortical thickness in prenatal mice [22]. Further research should explore if and how the splicing alterations in *NOTCH1* and its interactors affect the function of their protein products and determine their precise impact on the signaling pathway.

Given the major function of TDP-43 as a repressor of cryptic exon inclusion during RNA splicing [20], we specifically focused on the identification and study of cryptic transcripts in the FTLD-TDP patient cohort. In fact, 30 of the cryptic transcripts observed in the FCX of FTLD-TDP patients had a more than 10% increased expression as compared to controls. Twenty-eight of these were novel findings from this study, whereas two were previously reported as targets of TDP-43 loss of function, *STMN2* [61] and *ARHGAP32* [63]*. STMN2* encodes an axonal protein that has been extensively studied in the field of TDP-43 proteinopathies [9, 47, 61], while *ARHGAP32*, despite being identified in several studies, had not been highlighted for its relevance to the cryptic exon in this gene. ARHGAP32 (also known as RICS, p250GAP, p200RhoGAP) belongs to the Rho GTPase-activating proteins (RhoGAPs) family, and is a negative mediator of the Rho GTPases Cdc42, Rac1, and Rho [54] that plays a role in mediating axonal growth and regulation of spine morphogenesis [26, 31, 50]. Our data showed that the CEs in *STMN2* and *ARHGAP32* both demonstrated a unique and similar behavior (i) exhibiting more than 20% difference in expression between FTLD-TDP and controls in our short-read RNAseq dataset; (ii) correlating negatively with the age of onset in individuals with FTLD-TDP; (iii) showing a trend towards a negative correlation with *TARDBP* expression levels in neurons, which would indicate a potential linear relationship between TDP-43 loss of function and the induction of CEs; and (iv) being present in one (*ARHGAP32*) or both (*STMN2*) publicly available TDP-43 negative datasets [42, 63], reinforcing their consistent dysregulation across independent cohorts. This consistency, together with their correlation to disease phenotype (age of onset), strongly suggests these CEs are highly sensitive to TDP-43 loss of function. This result aligns with previous studies where it was shown that *STMN2* CE correlates with pTDP-43 and age of disease onset [61], and that it can be even more sensitive than pTDP-43 in detecting clinical phenotypes [65].

Focusing on the additional 28 cryptic events not previously reported in the literature, we found that 16 of these were also identified as differentially spliced in AD patients as compared to controls. This set of 16 cryptic events does not include *STMN2* and *ARHGAP32,* suggesting that these splicing alterations may not be driven by TDP-43 loss of function but instead implied the involvement of neurodegenerative processes shared between FTLD-TDP and AD, potentially extending to other neurodegenerative diseases. Most of these splicing events were identified in the TCX in AD, a region known to be one of the earliest brain regions affected [48]. Deep proteomics analyses in AD brains revealed an enrichment for RNA-binding proteins (RBPs) in hubs of differentially abundant proteins [30] that do not seem to be cell-type specific, as revealed in multiple single-nuclei RNAseq datasets [62]. In fact, some small nuclear ribonucleoproteins (snRNPs) have been observed to aggregate in AD and correlate with Aβ and neurofibrillary tangles [21]. Another family of RNA-binding proteins, the hnRNPs A1, A2B1 and K, mislocalize in several tauopathies, including FTLD-Tau and AD [33]. In FTLD, various RBPs besides TDP-43, particularly hnRNP K, exhibit nuclear depletion and mislocalization and induce cryptic exon inclusion [8, 28]. Based on these findings, we hypothesize that shared alterations in RBPs in AD and FTLD-TDP represent the underlying mechanism driving the significant splicing alterations identified in our study. In fact, common splicing changes between these two neurodegenerative diseases, together with FTLD-Tau and aged controls, were previously reported by Tollervey et al. [70]. In future studies, the list of 16 novel splicing alterations with marked differences between patients and controls in FTLD-TDP and AD identified in our study could offer novel perspectives into shared pathophysiological mechanisms underlying both neurodegenerative diseases.

Our findings emphasize *STMN2* and *ARHGAP32* as the strongest candidate biomarkers for both diagnosis and monitoring of FTLD-TDP progression. However, their potential translation into cryptic peptides has not yet been reported, contrarily to other known CE (*HDGFL2* and *ACTL6B*, among others) also identified in our dataset but with much smaller differences between FTLD-TDP patients and controls [63]. *STMN2* and *ARHGAP32* CEs are terminal exons that result in premature polyadenylation [14], potentially leading to truncated proteins or undergoing nonsense mediated decay (NMD) processes. Future research should prioritize investigating the translation of *STMN2* and *ARHGAP32* CEs into cryptic peptides to explore their possible role as diagnostic or disease progression biomarkers. Even if peptide translation is not detected, these CEs still hold significant potential as RNA biomarkers for clinical applications, whether cell-free or circulating in the CSF or plasma, or encapsulated within extracellular vesicles.

When we compared all cryptic transcripts significantly increased in our FTLD-TDP bulk brain dataset to controls (ΔPSI > 0 without a specific threshold) with cryptic transcripts identified in previous studies focused on TDP-43 KD iPSC-derived neurons and TDP-43 negative brain neuronal nuclei, 17 cryptic events were found to overlap with at least one previous study. In addition (and in contrast) to *STMN2* and *ARHGAP32*, this list included several cryptic transcripts with proven translation into cryptic peptides and detectability in the CSF of ALS patients [63]. Given the interest in these known TDP-43 targets, including *HDGFL2*, *ACTL6B*, and *KCNQ2*, we were surprised to detect only very subtle splicing alterations for these genes in brain tissue of our FTLD-TDP patients as compared to controls. This might be attributed to less expression of these genes in the brain or less sensitivity to TDP-43 loss of function. Recent studies have suggested that CEs and other cryptic splicing events are region-specific [53, 57], which could also explain the disparity observed across studies, especially in terms of magnitude of the change of the cryptic splicing event. It should also be considered that our cohort comprised FTLD-TDP patients with different pathological and genetical subtypes, with different degrees of pathology in the studied brain region (FCX), which adds heterogeneity and might have attenuated the differences between FTLD-TDP patients and controls. Regardless, with at most 1% difference in frontal cortex expression of cryptic transcripts between FTLD-TDP patients and controls for *HDGFL2*, *ACTL6B*, and *KCNQ2*, the development of a sensitive TDP-43 disease biomarker is expected to be challenging. In fact, neither *HDGFL2* nor *ACTL6B* transcripts was detected as significantly different when comparing TDP-43 positive with TDP-43 negative neuronal nuclei derived from human brain [42]. Combined with the expectation based on our work and others [2] that cryptic splicing events have different sensitivities to TDP-43 loss of function, future efforts may consider the development of a panel of cryptic peptides, rather than relying on a single target, for robust and accurate diagnosis of TDP-43 pathology and disease progression. Our catalog of TDP-43-driven cryptic splicing events observed in FTLD-TDP brain will provide a valuable foundation for these studies, paving the way for the development of more sensitive and specific diagnostic tools.

While our study provides valuable insights into splicing alterations in FTLD-TDP, it is important to acknowledge potential limitations. The use of short-read bulk RNA sequencing on brain tissue does not allow identification of cell-type-specific splicing changes and introduces the possibility of confounding due to variations in cell proportions associated with neurodegeneration. To mitigate these concerns, we adjusted for cell-type proportion differences in our DSA analysis, but this correction may not fully address the issue and could potentially lead to overcorrection. Future research should prioritize single-cell or single-nucleus RNA sequencing techniques, ideally coupled with long-read sequencing, to provide a more precise understanding of differentially spliced events within specific cell populations in FTLD-TDP. This approach could also expand the study of cryptic splicing to brain cell types beyond neurons, which were the primary focus of this and previous studies. In addition, the analysis of only one brain region has limited the comparison between FTLD-TDP subtypes, as some subtypes may exhibit greater pathology in other anatomical regions. A comprehensive analysis of multiple affected regions within the same individuals could provide deeper insights into subtype-specific differences and pathophysiological variations.

In conclusion, this study characterized the splicing landscape in FTLD-TDP, revealing distinct splicing patterns among different disease subtypes. Our findings contribute to the growing understanding that FTLD-TDP subtypes exhibit unique pathophysiological mechanisms, highlighting the importance of subtype-specific therapeutic strategies. We identified *STMN2* and *ARHGAP32* as particularly abundant and sensitive TDP-43-driven splicing events in brain tissue, along with an additional 16 novel neurodegeneration-driven splicing events that enhance our understanding of shared pathophysiological mechanisms between AD and FTLD-TDP. Furthermore, we compiled a catalog of TDP-43-associated splicing alterations observed across multiple studies, offering a valuable resource for biomarker research aimed at advancing diagnostic and therapeutic approaches for TDP-43 proteinopathies.

## Supplementary Information

Below is the link to the electronic supplementary material.

Supplementary file1 (DOCX 1806 KB)

Supplementary file2 (XLSX 12059 KB)

## Data Availability

Illumina short-read RNAseq data and corresponding sample metadata files are deposited in dbGaP, under the project title “RNA transcriptomic and DNA methylation landscape in FTLD-TDP and controls” (accession code phs004075.v1.p1). Additional information will be available upon reasonable request.

## References

[1] Ables JL, Breunig JJ, Eisch AJ, Rakic P (2011) Not(ch) just development: Notch signalling in the adult brain. Nat Rev Neurosci 12:269–283. 10.1038/nrn3024 21505516 10.1038/nrn3024 PMC3159580

[2] Agra Almeida Quadros AR, Li Z, Wang X, Ndayambaje IS, Aryal S, Ramesh N et al (2024) Cryptic splicing of stathmin-2 and UNC13A mRNAs is a pathological hallmark of TDP-43-associated Alzheimer’s disease. Acta Neuropathol. 10.1007/s00401-023-02655-0 38175301 10.1007/s00401-023-02655-0 PMC10766724

[3] Aleksander SA, Balhoff J, Carbon S, Cherry JM, Drabkin HJ, Ebert D et al (2023) The Gene Ontology knowledgebase in 2023. Genetics. 10.1093/genetics/iyad031 36866529 10.1093/genetics/iyad031 PMC10158837

[4] Allen M, Carrasquillo MM, Funk C, Heavner BD, Zou F, Younkin CS et al (2016) Human whole genome genotype and transcriptome data for Alzheimer’s and other neurodegenerative diseases. Sci Data. 10.1038/sdata.2016.89 27727239 10.1038/sdata.2016.89 PMC5058336

[5] Anders S, Pyl PT, Huber W (2015) HTSeq-A Python framework to work with high-throughput sequencing data. Bioinformatics 31:166–169. 10.1093/bioinformatics/btu638 25260700 10.1093/bioinformatics/btu638 PMC4287950

[6] Arseni D, Chen R, Murzin AG, Peak-Chew SY, Garringer HJ, Newell KL et al (2023) TDP-43 forms amyloid filaments with a distinct fold in type A FTLD-TDP. Nature 620:898–903. 10.1038/s41586-023-06405-w 37532939 10.1038/s41586-023-06405-w PMC10447236

[7] Arseni D, Nonaka T, Jacobsen MH, Murzin AG, Cracco L, Peak-Chew SY et al (2024) Heteromeric amyloid filaments of ANXA11 and TDP-43 in FTLD-TDP Type C. bioRxiv. 10.1101/2024.06.25.600403 39260416 10.1038/s41586-024-08024-5 PMC11485244

[8] Bampton A, Gatt A, Humphrey J, Cappelli S, Bhattacharya D, Foti S et al (2021) HnRNP K mislocalisation is a novel protein pathology of frontotemporal lobar degeneration and ageing and leads to cryptic splicing. Acta Neuropathol 142:609–627. 10.1007/s00401-021-02340-0 34274995 10.1007/s00401-021-02340-0 PMC8423707

[9] Baughn MW, Melamed Z, López-Erauskin J, Beccari MS, Ling K, Zuberi A et al (1979) (2023) Mechanism of STMN2 cryptic splice-polyadenylation and its correction for TDP-43 proteinopathies. Science 379:1140–1149. 10.1126/science.abq5622 10.1126/science.abq5622 PMC10148063 36927019

[10] Bennett DA, Buchman AS, Boyle PA, Barnes LL, Wilson RS, Schneider JA (2018) Religious orders study and rush memory and aging project. J Alzheimer’s Dis 64:S161–S189. 10.3233/JAD-179939 29865057 10.3233/JAD-179939 PMC6380522

[11] De Boer EMJ, Orie VK, Williams T, Baker MR, De Oliveira HM, Polvikoski T et al (2021) TDP-43 proteinopathies: a new wave of neurodegenerative diseases. J Neurol Neurosurg Psychiatry 92:86–95. 10.1136/jnnp-2020-322983 10.1136/jnnp-2020-322983 PMC7803890 33177049

[12] Bonham LW, Steele NZR, Karch CM, Broce I, Geier EG, Wen NL et al (2019) Genetic variation across RNA metabolism and cell death gene networks is implicated in the semantic variant of primary progressive aphasia. Sci Rep 9:10854. 10.1038/s41598-019-46415-1 31350420 10.1038/s41598-019-46415-1 PMC6659677

[13] Brown AL, Wilkins OG, Keuss MJ, Hill SE, Zanovello M, Lee WC et al (2022) TDP-43 loss and ALS-risk SNPs drive mis-splicing and depletion of UNC13A. Nature 603:131–137. 10.1038/s41586-022-04436-3 35197628 10.1038/s41586-022-04436-3 PMC8891020

[14] Bryce-Smith S, Brown A-L, Mehta PR, Mattedi F, Mikheenko A, Barattucci S et al (2024) TDP-43 loss induces extensive cryptic polyadenylation in ALS/FTD. bioRxiv. 10.1101/2024.01.22.576625 38313254 10.1101/2024.01.22.576625 PMC10836071

[15] Camacho C, Coulouris G, Avagyan V, Ma N, Papadopoulos J, Bealer K et al (2009) BLAST+: architecture and applications. BMC Bioinform. 10.1186/1471-2105-10-421 10.1186/1471-2105-10-421 PMC2803857 20003500

[16] Cotto KC, Feng YY, Ramu A, Richters M, Freshour SL, Skidmore ZL et al (2023) Integrated analysis of genomic and transcriptomic data for the discovery of splice-associated variants in cancer. Nat Commun 14:1589. 10.1038/s41467-023-37266-6 36949070 10.1038/s41467-023-37266-6 PMC10033906

[17] Davis SE, Cook AK, Hall JA, Voskobiynyk Y, Carullo NV, Boyle NR et al (2023) Patients with sporadic FTLD exhibit similar increases in lysosomal proteins and storage material as patients with FTD due to GRN mutations. Acta Neuropathol Commun 11:70. 10.1186/s40478-023-01571-4 37118844 10.1186/s40478-023-01571-4 PMC10148425

[18] Dickson DW, Baker MC, Jackson JL, Dejesus-Hernandez M, Finch NCA, Tian S et al (2019) Extensive transcriptomic study emphasizes importance of vesicular transport in C9orf72 expansion carriers. Acta Neuropathol Commun 7:150. 10.1186/s40478-019-0797-0 31594549 10.1186/s40478-019-0797-0 PMC6781370

[19] Dobin A, Davis CA, Schlesinger F, Drenkow J, Zaleski C, Jha S et al (2013) STAR: Ultrafast universal RNA-seq aligner. Bioinformatics 29:15–21. 10.1093/bioinformatics/bts635 23104886 10.1093/bioinformatics/bts635 PMC3530905

[20] Donde A, Sun M, Ling JP, Braunstein KE, Pang B, Wen X et al (2019) Splicing repression is a major function of TDP-43 in motor neurons. Acta Neuropathol 138:813–826. 10.1007/s00401-019-02042-8 31332509 10.1007/s00401-019-02042-8 PMC6802294

[21] Hales CM, Dammer EB, Deng Q, Duong DM, Gearing M, Troncoso JC et al (2016) Changes in the detergent-insoluble brain proteome linked to amyloid and tau in Alzheimer’s Disease progression. Proteomics 16:3042–3053. 10.1002/pmic.201600057 27718298 10.1002/pmic.201600057 PMC5462625

[22] Hendricks E, Quihuis AM, Hung ST, Chang J, Dorjsuren N, Der B et al (2023) The C9ORF72 repeat expansion alters neurodevelopment. Cell Rep 42:112983. 10.1016/j.celrep.2023.112983 37590144 10.1016/j.celrep.2023.112983 PMC10757587

[23] Hofmann JW, Seeley WW, Huang EJ (2019) RNA binding proteins and the pathogenesis of frontotemporal lobar degeneration. Annu Rev Pathol 14:469–495. 10.1146/annurev-pathmechdis-012418-012955 30355151 10.1146/annurev-pathmechdis-012418-012955 PMC6731550

[24] Humphrey J, Emmett W, Fratta P, Isaacs AM, Plagnol V (2017) Quantitative analysis of cryptic splicing associated with TDP-43 depletion. BMC Med Genomics 10:38. 10.1186/s12920-017-0274-1 28549443 10.1186/s12920-017-0274-1 PMC5446763

[25] Humphrey J, Knowles DA, Li YI (2021) LeafViz

[26] Impey S, Davare M, Lasiek A, Fortin D, Ando H, Varlamova O et al (2010) An activity-induced microRNA controls dendritic spine formation by regulating Rac1-PAK signaling. Mol Cell Neurosci 43:146–156. 10.1016/j.mcn.2009.10.005 19850129 10.1016/j.mcn.2009.10.005 PMC2818337

[27] Irwin KE, Jasin P, Braunstein KE, Sinha IR, Garret MA, Bowden KD et al (2024) A fluid biomarker reveals loss of TDP-43 splicing repression in presymptomatic ALS–FTD. Nat Med 30:382–393. 10.1038/s41591-023-02788-5 38278991 10.1038/s41591-023-02788-5 PMC10878965

[28] Jiang X, Gatt A, Lashley T (2023) HnRNP pathologies in frontotemporal lobar degeneration. Cells 12:1633. 10.3390/cells12121633 37371103 10.3390/cells12121633 PMC10297297

[29] Jo M, Lee S, Jeon YM, Kim S, Kwon Y, Kim HJ (2020) The role of TDP-43 propagation in neurodegenerative diseases: integrating insights from clinical and experimental studies. Exp Mol Med 52:1652–1662. 10.1038/s12276-020-00513-7 33051572 10.1038/s12276-020-00513-7 PMC8080625

[30] Johnson ECB, Dammer EB, Duong DM, Yin L, Thambisetty M, Troncoso JC et al (2018) Deep proteomic network analysis of Alzheimer’s disease brain reveals alterations in RNA binding proteins and RNA splicing associated with disease. Mol Neurodegener 13:52. 10.1186/s13024-018-0282-4 30286791 10.1186/s13024-018-0282-4 PMC6172707

[31] Kannan M, Lee SJ, Schwedhelm-Domeyer N, Nakazawa T, Stegmüller J (2012) p250GAP Is a novel player in the Cdh1-APC/Smurf1 pathway of axon growth regulation. PLoS ONE 7:e50735. 10.1371/journal.pone.0050735 23226367 10.1371/journal.pone.0050735 PMC3511349

[32] Kassambara A (2023) ggpubr: “ggplot2” Based Publication Ready Plots. R package version 0.6.0

[33] Kavanagh T, Balcomb K, Ahmadi Rastegar D, Lourenco GF, Wisniewski T, Halliday G et al (2024) hnRNP A1, hnRNP A2B1, and hnRNP K are dysregulated in tauopathies, but do not colocalize with tau pathology. Brain Pathol. 10.1111/bpa.13305 39354671 10.1111/bpa.13305 PMC11961206

[34] Kolde R (2018) pheatmap: pretty heatmaps. R package version 1:12

[35] Li H (2021) New strategies to improve minimap2 alignment accuracy. Bioinformatics 37:4572–4574. 10.1093/bioinformatics/btab705 34623391 10.1093/bioinformatics/btab705 PMC8652018

[36] Li YI, Knowles DA, Humphrey J, Barbeira AN, Dickinson SP, Im HK et al (2018) Annotation-free quantification of RNA splicing using LeafCutter. Nat Genet 50:151–158. 10.1038/s41588-017-0004-9 29229983 10.1038/s41588-017-0004-9 PMC5742080

[37] Liao YZ, Ma J, Dou JZ (2022) The role of TDP-43 in neurodegenerative disease. Mol Neurobiol 59:4223–4241. 10.1007/s12035-022-02847-x 35499795 10.1007/s12035-022-02847-x

[38] Licht-Murava A, Meadows SM, Palaguachi F, Song SC, Jackvony S, Bram Y et al (2023) Astrocytic TDP-43 dysregulation impairs memory by modulating antiviral pathways and interferon-inducible chemokines. Sci Adv 9:eade1282. 10.1126/sciadv.ade1282 37075107 10.1126/sciadv.ade1282 PMC10115456

[39] Ling JP, Pletnikova O, Troncoso JC, Wong PC (2015) TDP-43 repression of nonconserved cryptic exons is compromised in ALS-FTD. Science 349:650–655. 10.1126/science.aab0983 26250685 10.1126/science.aab0983 PMC4825810

[40] Livak KJ, Schmittgen TD (2001) Analysis of relative gene expression data using real-time quantitative PCR and the 2-ΔΔCT method. Methods 25:402–408. 10.1006/meth.2001.1262 11846609 10.1006/meth.2001.1262

[41] Love MI, Huber W, Anders S (2014) Moderated estimation of fold change and dispersion for RNA-seq data with DESeq2. Genome Biol. 10.1186/s13059-014-0550-8 25516281 10.1186/s13059-014-0550-8 PMC4302049

[42] Ma XR, Prudencio M, Koike Y, Vatsavayai SC, Kim G, Harbinski F et al (2022) TDP-43 represses cryptic exon inclusion in the FTD–ALS gene UNC13A. Nature 603:124–130. 10.1038/s41586-022-04424-7 35197626 10.1038/s41586-022-04424-7 PMC8891019

[43] Mackenzie IR, Neumann M (2017) Reappraisal of TDP-43 pathology in FTLD-U subtypes. Acta Neuropathol 134:79–96. 10.1007/s00401-017-1716-8 28466142 10.1007/s00401-017-1716-8

[44] Mackenzie IRA, Neumann M, Baborie A, Sampathu DM, Du Plessis D, Jaros E et al (2011) A harmonized classification system for FTLD-TDP pathology. Acta Neuropathol 122:111–113. 10.1007/s00401-011-0845-8 21644037 10.1007/s00401-011-0845-8 PMC3285143

[45] McKenzie AT, Wang M, Hauberg ME, Fullard JF, Kozlenkov A, Keenan A et al (2018) Brain cell type specific gene expression and co-expression network architectures. Sci Rep 8:8868. 10.1038/s41598-018-27293-5 29892006 10.1038/s41598-018-27293-5 PMC5995803

[46] Mehta PR, Brown AL, Ward ME, Fratta P (2023) The era of cryptic exons: implications for ALS-FTD. Mol Neurodegener 18:16. 10.1186/s13024-023-00608-5 36922834 10.1186/s13024-023-00608-5 PMC10018954

[47] Melamed Z, López-Erauskin J, Baughn MW, Zhang O, Drenner K, Sun Y et al (2019) Premature polyadenylation-mediated loss of stathmin-2 is a hallmark of TDP-43-dependent neurodegeneration. Nat Neurosci 22:180–190. 10.1038/s41593-018-0293-z 30643298 10.1038/s41593-018-0293-z PMC6348009

[48] Migliaccio R, Cacciamani F (2022) The temporal lobe in typical and atypical Alzheimer disease. Handb Clin Neurol 187:449–466. 10.1016/B978-0-12-823493-8.00004-3 35964987 10.1016/B978-0-12-823493-8.00004-3

[49] Mostafavi S, Gaiteri C, Sullivan SE, White CC, Tasaki S, Xu J et al (2018) A molecular network of the aging human brain provides insights into the pathology and cognitive decline of Alzheimer’s disease. Nat Neurosci 21:811–819. 10.1038/s41593-018-0154-9 29802388 10.1038/s41593-018-0154-9 PMC6599633

[50] Nasu-Nishimura Y, Hayashi T, Ohishi T, Okabe T, Ohwada S, Hasegawa Y et al (2006) Role of the Rho GTPase-activating protein RICS in neurite outgrowth. Genes Cells 11:607–614. 10.1111/j.1365-2443.2006.00966.x 16716191 10.1111/j.1365-2443.2006.00966.x

[51] Neumann M, Kwong LK, Truax AC, Vanmassenhove B, Kretzschmar HA, Van Deerlin VM et al (2007) TDP-43-positive white matter pathology in frontotemporal lobar degeneration with ubiquitin-positive inclusions. J Neuropathol Exp Neurol 66:177–83. 10.1097/01.jnen.0000248554.45456.58 17356379 10.1097/01.jnen.0000248554.45456.58

[52] Neumann M, Lee EB, Mackenzie IR (2021) Frontotemporal lobar degeneration TDP-43-immunoreactive pathological subtypes: clinical and mechanistic significance. Adv Exp Med Biol 1281:201–217. 10.1007/978-3-030-51140-1_13 33433877 10.1007/978-3-030-51140-1_13 PMC8183578

[53] Newton DF, Yang R, Gutierrez J, Hofmann JW, Yeh FL, Biever A et al (2024) TDP43 proteinopathy exhibits disease, tissue, and context-specific cryptic splicing signatures. bioRxiv. 10.1101/2024.03.29.58724439677800

[54] Okabe T, Nakamura T, Nishimura YN, Kohu K, Ohwada S, Morishita Y et al (2003) RICS, a novel GTPase-activating protein for Cdc42 and Rac1, is involved in the β-Catenin-N-cadherin and N-methyl-D-aspartate receptor signaling. J Biol Chem 278:9920–9927. 10.1074/jbc.M208872200 12531901 10.1074/jbc.M208872200

[55] Olney NT, Spina S, Miller BL (2017) Frontotemporal dementia. Neurol Clin 35:339–374. 10.1016/j.ncl.2017.01.008 28410663 10.1016/j.ncl.2017.01.008 PMC5472209

[56] Pagès H, Aboyoun P, Gentleman R, DebRoy S (2024) Biostrings: Efficient manipulation of biological strings

[57] Pickles SR, Gonzalez Bejarano J, Narayan A, Daughrity L, Maroto Cidfuentes C, Reeves MM et al (2025) TDP-43 cryptic RNAs in perry syndrome: differences across brain regions and TDP-43 proteinopathies. Mov Disord. 10.1002/mds.30104 39788898 10.1002/mds.30104 PMC12006891

[58] Pottier C, Küçükali F, Baker M, Batzler A, Jenkins GD, van Blitterswijk M et al (2024) Deciphering distinct genetic risk factors for FTLD-TDP pathological subtypes via whole-genome sequencing. medRxiv. 10.1101/2024.06.24.24309088 40280976 10.1038/s41467-025-59216-0 PMC12032271

[59] Pottier C, Mateiu L, Baker MC, Dejesus-Hernandez M, Teixeira Vicente C, Finch NA et al (2022) Shared brain transcriptomic signature in TDP-43 type A FTLD patients with or without GRN mutations. Brain 145:2472–2485. 10.1093/brain/awab437 34918030 10.1093/brain/awab437 PMC9337811

[60] Prjibelski AD, Mikheenko A, Joglekar A, Smetanin A, Jarroux J, Lapidus AL et al (2023) Accurate isoform discovery with IsoQuant using long reads. Nat Biotechnol 41:915–918. 10.1038/s41587-022-01565-y 36593406 10.1038/s41587-022-01565-y PMC10344776

[61] Prudencio M, Humphrey J, Pickles S, Brown AL, Hill SE, Kachergus JM et al (2020) Truncated stathmin-2 is a marker of TDP-43 pathology in frontotemporal dementia. J Clin Investig 130:6080–6092. 10.1172/JCI139741 32790644 10.1172/JCI139741 PMC7598060

[62] Rybak-Wolf A, Plass M (2021) RNA dynamics in Alzheimer’s Disease. Molecules 26:5113. 10.3390/molecules26175113 34500547 10.3390/molecules26175113 PMC8433936

[63] Seddighi S, Qi YA, Brown A-L, Wilkins OG, Bereda C, Belair C et al (2024) Mis-spliced transcripts generate de novo proteins in TDP-43-related ALS/FTD. Sci Transl Med 16:7162. 10.1126/scitranslmed.adg7162 10.1126/scitranslmed.adg7162 PMC11325748 38277467

[64] Shi Y, Kirwan P, Livesey FJ (2012) Directed differentiation of human pluripotent stem cells to cerebral cortex neurons and neural networks. Nat Protoc 7:1836–1846. 10.1038/nprot.2012.116 22976355 10.1038/nprot.2012.116

[65] Spence H, Waldron FM, Saleeb RS, Brown AL, Rifai OM, Gilodi M et al (2024) RNA aptamer reveals nuclear TDP-43 pathology is an early aggregation event that coincides with STMN-2 cryptic splicing and precedes clinical manifestation in ALS. Acta Neuropathol 147:50. 10.1007/s00401-024-02705-1 38443601 10.1007/s00401-024-02705-1 PMC10914926

[66] Spinelli EG, Mandelli ML, Miller ZA, Santos-Santos MA, Wilson SM, Agosta F et al (2017) Typical and atypical pathology in primary progressive aphasia variants. Ann Neurol 81:430–443. 10.1002/ana.24885 28133816 10.1002/ana.24885 PMC5421819

[67] Szklarczyk D, Kirsch R, Koutrouli M, Nastou K, Mehryary F, Hachilif R et al (2023) The STRING database in 2023: protein-protein association networks and functional enrichment analyses for any sequenced genome of interest. Nucleic Acids Res 51:D638–D646. 10.1093/nar/gkac1000 36370105 10.1093/nar/gkac1000 PMC9825434

[68] Team TBD (2023) BSgenome.Hsapiens.UCSC.hg38: Full genomic sequences for Homo sapiens (UCSC genome hg38)

[69] Thorvaldsdóttir H, Robinson JT, Mesirov JP (2013) Integrative genomics viewer (IGV): High-performance genomics data visualization and exploration. Brief Bioinform 14:178–192. 10.1093/bib/bbs017 22517427 10.1093/bib/bbs017 PMC3603213

[70] Tollervey JR, Wang Z, Hortobágyi T, Witten JT, Zarnack K, Kayikci M et al (2011) Analysis of alternative splicing associated with aging and neurodegeneration in the human brain. Genome Res 21:1572–1582. 10.1101/gr.122226.111 21846794 10.1101/gr.122226.111 PMC3202275

[71] Udine E, DeJesus-Hernandez M, Tian S, Das Neves SP, Crook R, Finch NCA et al (2024) Abundant transcriptomic alterations in the human cerebellum of patients with a C9orf72 repeat expansion. Acta Neuropathol 147:73. 10.1007/s00401-024-02720-2 38641715 10.1007/s00401-024-02720-2 PMC11031479

[72] Vialle RA, de Paiva LK, Bennett DA, Crary JF, Raj T (2022) Integrating whole-genome sequencing with multi-omic data reveals the impact of structural variants on gene regulation in the human brain. Nat Neurosci 25:504–514. 10.1038/s41593-022-01031-7 35288716 10.1038/s41593-022-01031-7 PMC9245608

[73] Wang L, Wang S, Li W (2012) RSeQC: quality control of RNA-seq experiments. Bioinformatics 28:2184–2185. 10.1093/bioinformatics/bts356 22743226 10.1093/bioinformatics/bts356

[74] Wang M, Beckmann ND, Roussos P, Wang E, Zhou X, Wang Q et al (2018) The Mount Sinai cohort of large-scale genomic, transcriptomic and proteomic data in Alzheimer’s disease. Sci Data 5:180185. 10.1038/sdata.2018.185 30204156 10.1038/sdata.2018.185 PMC6132187

[75] Wickham H (2016) ggplot2: Elegant graphics for data analysis. 10.1007/978-0-387-98141-3

[76] Wu R, Ye Y, Dong D, Zhang Z, Wang S, Li Y et al (2024) Disruption of nuclear speckle integrity dysregulates RNA splicing in C9ORF72-FTD/ALS. Neuron 112:3434–3451. 10.1016/j.neuron.2024.07.025 39181135 10.1016/j.neuron.2024.07.025 PMC11502262

[77] Wu T, Hu E, Xu S, Chen M, Guo P, Dai Z et al (2021) clusterProfiler 4.0: a universal enrichment tool for interpreting omics data. Innovation 2:100141. 10.1016/j.xinn.2021.100141 34557778 10.1016/j.xinn.2021.100141 PMC8454663

[78] Yang D, Abdallah A, Li Z, Lu Y, Almeida S, Gao FB (2015) FTD/ALS-associated poly(GR) protein impairs the Notch pathway and is recruited by poly(GA) into cytoplasmic inclusions. Acta Neuropathol 130:525–535. 10.1007/s00401-015-1448-6 26031661 10.1007/s00401-015-1448-6 PMC4575383

[79] Yu G, Hu E, Gao C-H (2023) enrichplot: Visualization of Functional Enrichment Result. R package version 1.22.0

[80] Zhao W, Zhang S, Zhu Y, Xi X, Bao P, Ma Z et al (2022) POSTAR3: An updated platform for exploring post-transcriptional regulation coordinated by RNA-binding proteins. Nucleic Acids Res 50:D287–D294. 10.1093/nar/gkab702 34403477 10.1093/nar/gkab702 PMC8728292

[81] Zhong Y, Wan Y-W, Pang K, Chow LM, Liu Z (2013) Digital sorting of complex tissues for cell type-specific gene expression profiles. BMC Bioinform 7(14):89. 10.1186/1471-2105-14-89 10.1186/1471-2105-14-89 PMC3626856 23497278

