# Supplementary material for: Analysis of the splicing landscape of the frontal cortex in FTLD-TDP reveals subtype specific patterns and cryptic splicing
Source: Acta Neuropathol. 2025 Jun 6;149(1):59. doi: 10.1007/s00401-025-02901-7 (PMC12143990; doi:10.1007/s00401-025-02901-7)
Supplement: Supplementary file 1 — Supplementary file1 (DOCX 1806 KB) [file 401_2025_2901_MOESM1_ESM.docx]

**
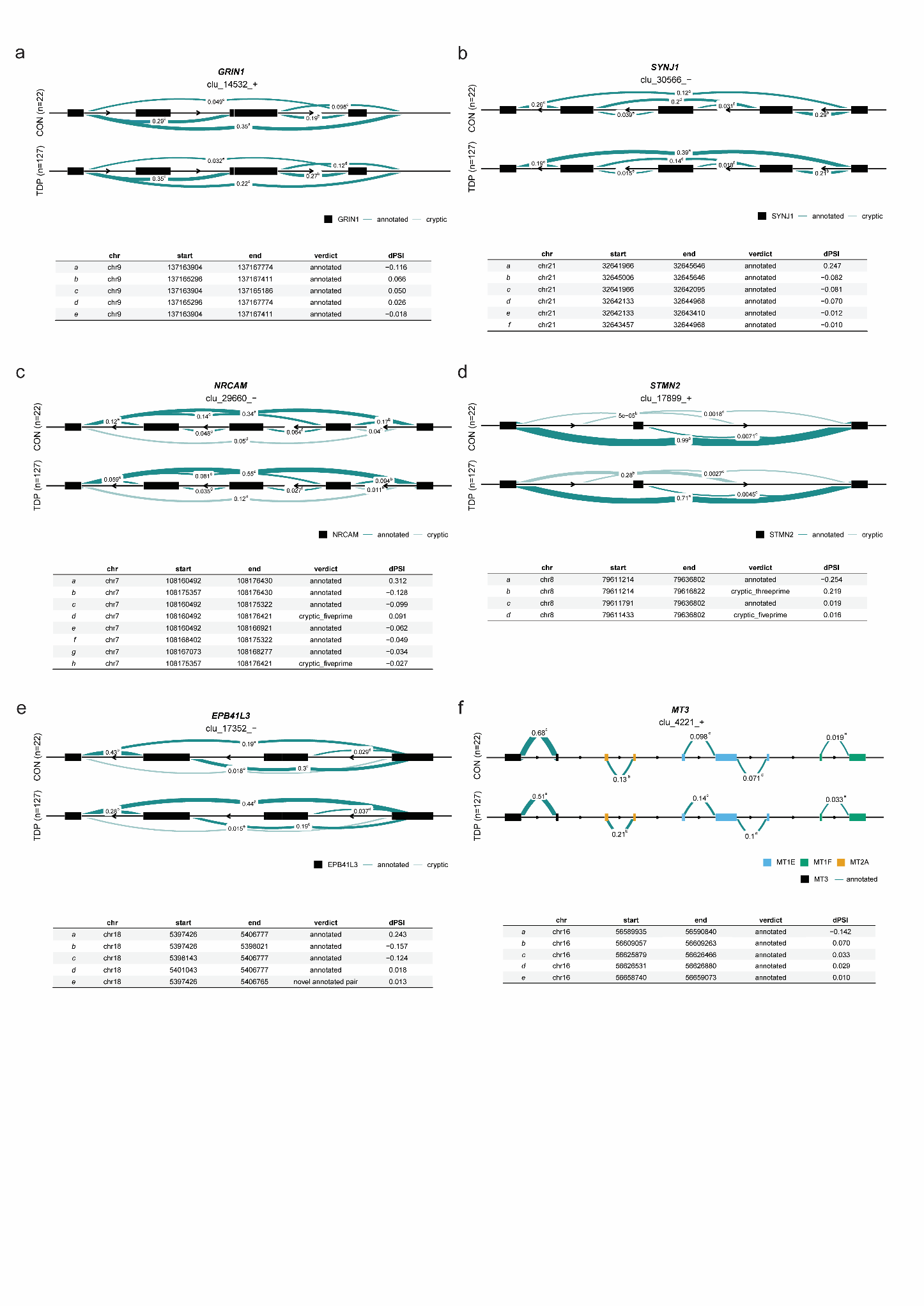
**

**Figure S1. Sashimi plots of differentially spliced clusters in FTLD-TDP. (a)** *GRIN1*. **(b)** *SYNJ1.* **(c)** *NRCAM.* **(d)** *STMN2* **(e)** *EPB41L3.* **(f)** *MT3.* Light green lines represent cryptic events, dark green lines represent annotated events. The verdict column indicates the status of the event: annotated, cryptic five prime (novel donor), cryptic three prime (novel acceptor) or novel annotated pair (novel donor-acceptor combination). TDP: FTLD-TDP; CON: Control; PSI: Percent Spliced in Index.


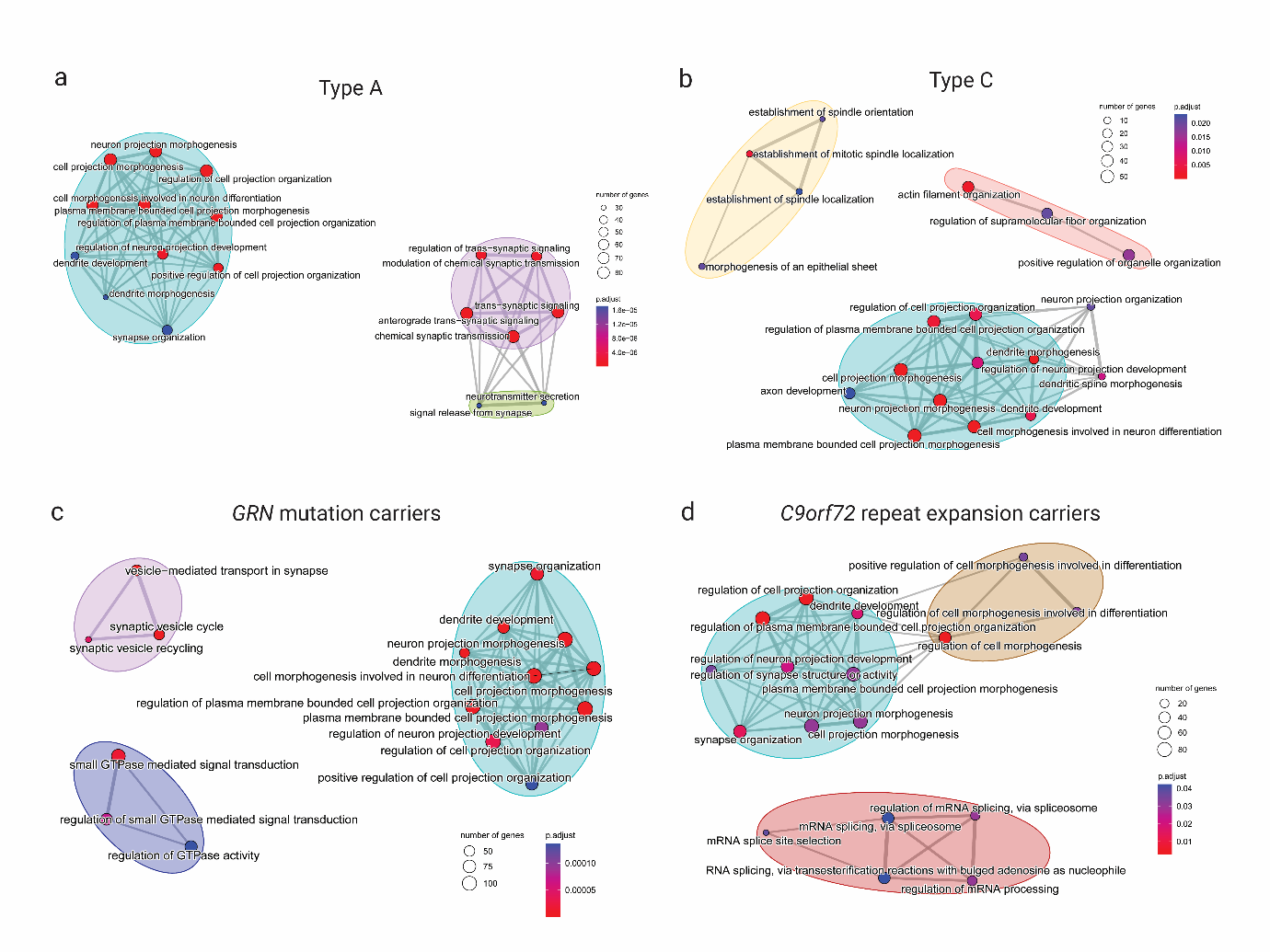


**Figure S2. Over-representation pathway analysis (ORA) in FTLD-TDP subtypes. (a)** ORA for FTLD-TDP Type A analysis. **(b)** ORA for FTLD-TDP Type C analysis. **(c)** ORA for FTLD-TDP *GRN*-mutation carriers’ analysis. **(d)** ORA for FTLD-TDP *C9orf72* repeat expansion carriers’ analysis. Data without cell proportions adjustment was analyzed. Each network represents the top 20 most dysregulated pathways, but modules with only one node were excluded. The size of the circles represents the number of genes undergoing differential splicing in that specific enriched pathway. The color of the circles represents the adjusted p-values. Same colors among networks represent similar clusters of pathways.


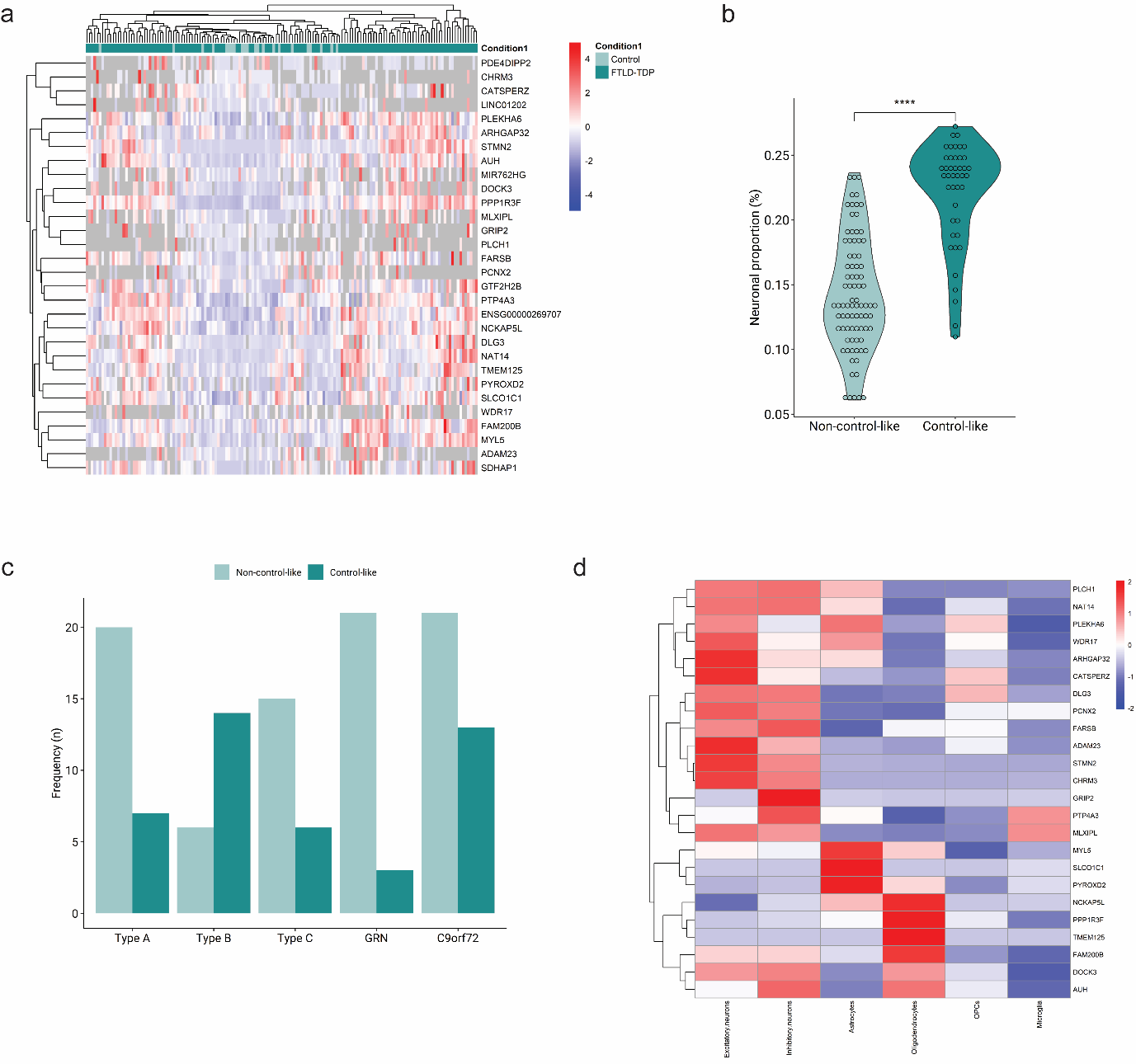


**Figure S3. Genes with cryptic splicing in FTLD-TDP. (a)** Heatmap representing Percent Spliced in Index (PSI) values per subject for each of the 30 genes showing cryptic events with more than 10% difference in expression between FTLD-TDP patients and controls. Values were scaled per column, and PSI values with less than 5 counts in the event were removed from the analysis. Light green represents controls and dark green represents patients. (b) Neuronal proportion of FTLD-TDP patients that cluster with controls (control-like) and those that do not cluster (non-control-like). Unpaired t-test, ****p<0.0001. **(c)** Frequency of control-like FTLD-TDP patients in each FTLD-TDP subgroup. Chi-square test, p=0.001. **(d)** Heatmap representing the gene expression (nTPMs) of the selected 30 candidates in each main brain cell type. Data was extracted from The Human Protein Altas. Values were scaled per row.

**
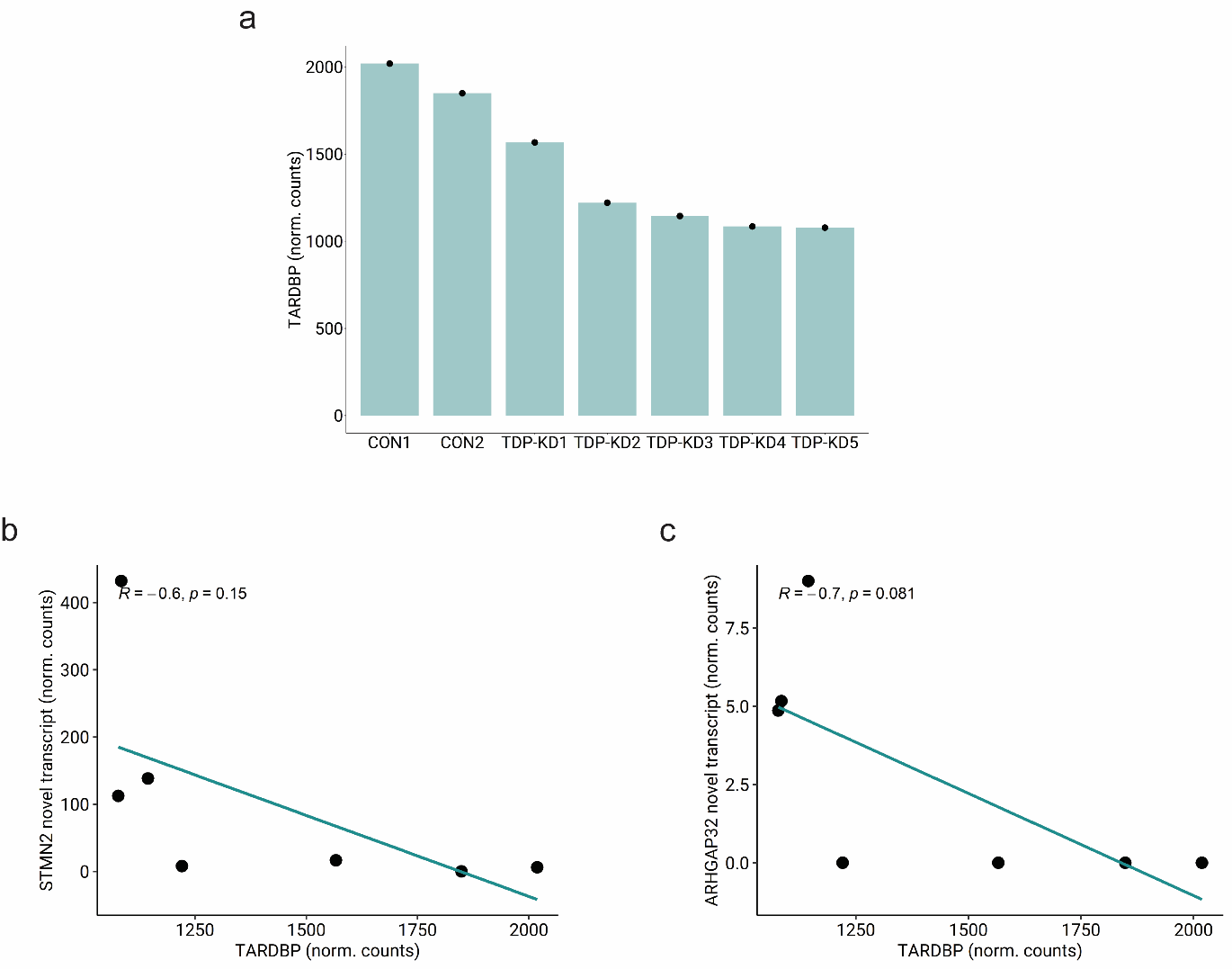
**

**Figure S4. *TARDBP* KD in iPSC-derived neurons. (a)** *TARDBP* expression (normalized counts) of the iPSC-derived neurons, measured by long-read cDNA sequencing. **(b)** Scatter plot representing the expression of the novel transcript with the cryptic exon in *STMN2* (y-axis) and *TARDBP* expression in iPSC-derived neurons (x-axis). Spearman correlation. **(c)** Scatter plot representing the overall expression of transcripts with the cryptic exon in *ARHGAP32* (y-axis) and *TARDBP* expression in iPSC-derived neurons (x-axis). Spearman correlation.

**
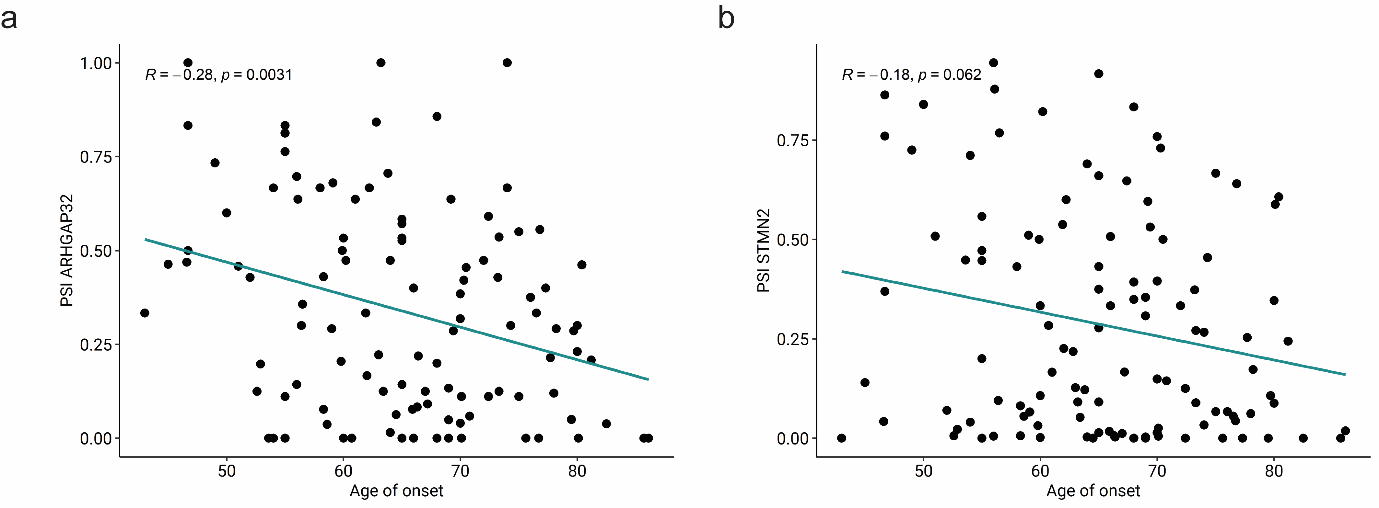
**

**Figure 5. Correlation between the age of disease onset and cryptic splicing.** Percent Spliced in Index (PSI) values with less than 5 counts in the event were removed from the analysis. **(a)** Scatter plot representing the PSI of *ARHGAP32* CE from the short-read RNAseq analysis (y-axis) and age of onset of the disease (x-axis) of all FTLD-TDP patients. Spearman correlation. **(b)** Scatter plot representing the PSI of *STMN2* CE from the short-read RNAseq analysis (y-axis) and age of onset of the disease (x-axis) of all FTLD-TDP patients. Spearman correlation.
